# Lessons learned from a structured and descriptive analysis of nosocomial outbreaks in hematology-oncology

**DOI:** 10.1186/s12879-026-14279-2

**Published:** 2026-08-20

**Authors:** Sara-Lena Posselt, Claas Baier, Frank Schwab, Ralf-Peter Vonberg

**Affiliations:** 1 Institute for Medical Microbiology and Hospital Epidemiology, Hannover Medical School, Carl-Neuberg-Str. 1, D-30625 Hannover, Germany; 2 Institute for Hygiene and Environmental Health, Charité – University Medicine Berlin, Berlin, Germany; 3 Infection Control Department, Agaplesion Diakonieklinikum Rotenburg, Rotenburg, Germany

**Keywords:** Hematology and oncology, Review, Pathogen transmission, Nosocomial outbreak, Infection control

## Abstract

Nosocomial outbreaks among immunocompromised patients are serious events. We conducted a descriptive analysis of nosocomial outbreaks in hematology and oncology, drawing on data from 196 outbreaks included in the Worldwide Outbreak Database. Our analysis includes patient characteristics, pathogens, outbreak sources, routes of transmission, and infection control measures. Furthermore, it determines risk factors for increased infection control effort by using the Weighted Cumulative Hygiene Score (WCHS). Bloodstream infections and lower respiratory tract infections were most often observed in the outbreak patient population. The primary infectious agents were Pseudomonas aeruginosa and other non-fermenting Gram-negative rods as well as enterococci. An index person (patient/staff) and the environment were the main reservoirs for transmission. Noteworthy, the exact source could not be identified in nearly half of the outbreak events. Infection control measures were usually implemented as bundles including but not limited to screening of patients, changes in the disinfection or sterilization regime and enforcement of proper hand hygiene. Multivariable regression analysis revealed the following factors being independently associated with a high infection control effort: causative agent fungi (other than molds) (OR=6.26; CI95:1.60–24.44), multi-drug resistant bacteria (OR=6.01; CI95:2.56–14.09), antibiotic therapy (OR=2.97; CI95:1.05–8.38), a high number (≥ 10) of patients involved in the outbreak (OR=2.54; CI95:1.11–5.81), and the transmission route being direct contact (OR=3.42; CI95:1.43–8.16). The occurrence of lower respiratory tract infections was strongly associated with mortality (OR=7.95; CI95:2.06–30.73). Staff working in hematology and oncology should be aware of these details in order to approach a nosocomial outbreak appropriately at a most early time point.

**Supplementary Information:**

The online version contains supplementary material available at 10.1186/s12879-026-14279-2.

## Introduction

Nosocomial outbreaks (NOs) of infectious diseases can occur anytime on any ward in any medical specialty [1–3]. Such an event always poses a serious threat to the ward and the patients concerned, particularly if those patients are immunocompromised. In this context, a hematology and oncology (H/O) unit contains one of the most vulnerable patient populations due to the underlying disease and immunosuppressive anti-cancer treatment [4, 5].

There are numerous publications (outbreak reports) on NOs occurring on H/O wards [6, 7], but analyses of these events usually focus on only one specific type of pathogen [8, 9]. Thus, data on a broader view of NOs in H/O are scarce. Nonetheless, the insights derived from NOs are of paramount importance for guiding future NO investigations. In addition, they augment the continuous education of staff and aid the development of adapted and tailored infection control guidelines in H/O wards [10]. To better understand the role of NO investigation, we conducted a descriptive, structured analysis of published NOs in H/O – the largest of its kind to the best of our knowledge.

## Materials and methods

### Data sources and definitions

The source of published articles on NOs for this analysis was the Worldwide Outbreak Database (https://www.outbreak-database.com), which represents the today´s largest collection of all types of NOs [11, 12]. This database provided the various characteristics of NOs in an easy-to-use standardized and categorized manner. It comprised articles – outbreak reports or reports containing nosocomial clusters – as published by the primary authors, but once more assessed by the editorial team of the database itself. Across the included literature, there was heterogenicity regarding the definition of a NO. “Events with epidemiologically related nosocomial acquisition of a pathogen by at least two patients” served as a robust core feature of all articles. This encompassed both infections and horizontal transmission leading to colonization only. All NOs published in English language related to H/O departments were included for further investigation and reviewed in detail in the full text. The search covered articles published between 1969 and 2020.

### Data extraction

We assessed the following parameters of each outbreak:

- Setting: Year, country, number of affected wards including intensive care units (ICU), and overall NO duration.
- Patients: Differentiation between hematology and oncology including the underlying malignancy, age, and gender.
- Clinical outcome: Number and types of infection among patients vs. colonization only as well as the mortality.
- Pathogen: Genus, species, type of multi drug resistance (if applicable), the source, and the route of transmission.
- Infection control (IC) measures: Type of newly implemented and/or enforced infection control measures such as changes in disinfection and/or sterilization procedures or screening of patients (see Table 1).

**Table 1:** Weighted Cumulative Hygiene Score (WCHS) [13]

Item	Infection control measures	Score
1	Screening of staff	3
2	Closure of the ward/department	3
3	Improvement of staff-to-patient ratio or other changes in staffing	3
4	Screening of environment (including for instance air, water, surfaces)	2
5	Changes in disinfection and/or sterilization procedures	2
6	Changes in handling of medical devices or technical equipment	2
7	Screening of patients	1
8	Isolation (single room or cohort)	1
9	Change of antibiotic therapy, prophylaxis or vaccination	1
10	Improvement of hand hygiene	1
11	Improvement of protective clothing	1
12	Training and education of staff	1
	**Maximum score**	**21**

Scoring reflects the infection control effort attributed to a specific infection control measure

If data or details were missing in an NO report those items could not be included for the respective analysis and the calculation of corresponding parameters such as total number or median.

### Assessment of infection control measures with the Weighted Cumulative Hygiene Score (WCHS)

Not all IC measures were equally challenging; some were more difficult to implement and significantly more costly than others. To better quantify the actual IC effort, we evaluated all explicitly mentioned IC measures using the established WCHS, as described in detail before [13] and in Table 1. This score was the result of an expert consensus considering clinical experience in both hospital epidemiology and infectious diseases, and quantified the intensity of the response to a NO. Each measure was assigned a weight (1, 2 or 3) reflecting the complexity and resource intensity of its implementation. The sum of the WCHS ranged from 0, indicating no changes in IC measures, up to a maximum of 21, if all possible IC measures were implemented within the NO.

### Statistical analyses

In the descriptive analysis of the NOs, number and percent or median and interquartile range (IQR) were calculated. Differences were tested by Chi-square test or Wilcoxon rank-sum test. The former was used for categorical comparisons whereas the latter was used for comparison of continuous variable distributions. To identify (risk) factors for a high (score > 7), moderate (score > 4) and low (score = 0) WCHS and for the outcome “patients died within the outbreak (mortality)”, univariable and multivariable regression analyses were performed using logistic regression models. For this, parameters were transformed in dummy or categorical parameters as appropriate. Parameters with missing values were categorized accordingly. For example, the number of patients involved in a NO was categorized as ≥ 10, <10, or not specified (n. s.). Odds ratios (ORs) were calculated by comparing each category to a reference category. In this example, ≥ 10 versus < 10 patients involved in an NO, or, in other cases, “yes” versus “no”. All observations, including outbreaks with an unspecified number of patients involved, were included in the models. Selected parameters (such as type of pathogen and infections observed, multidrug resistance, suspected transmission route or the outbreak size) which presumably influence the WCHS and mortality were investigated in the regression analysis. The following parameters were considered: size of the NO, persons affected in the NO, comorbidities, treatment and devices, groups of causative agents in NO, types of causative agents in NO, routes of transmission in NO, types of infections in NO, multi-drug resistant pathogens involved in NO, sources of NO and causes of NO (Supplemental Tables A, B and C).

A multivariable forward stepwise regression was performed with entry and retention criteria (p-values) set at *p* ≤ 0.05 and *p* < 0.055, respectively. P-values less than 0.05 were considered significant. All analyses were exploratory in manor performed using SPSS (IBM SPSS statistics, Somer, NY, USA) and SAS 9.4 (SAS Institute, Cary, NC, USA).

## Results

### Articles

There were 196 NOs published from the year 1969 up to the year 2020 that qualified for inclusion in this analysis. The majority of the articles were from Europe (102 NOs; 52.0%) followed by North America (47 NOs; 24.0%) and Asia (27 NOs; 13.8%). A list of all articles included is provided as Supplemental Table D.

### Outbreak and patient characteristics

The median NO duration was 18 weeks (IQR: 8–52); 19 reports did not provide explicit information on duration. In 15 NO (7.7%), intensive care units were affected. Overall, there were 3,809 patients affected of which 2,955 (77.6%) had a specific hematological-oncological underlying disease. A median of 11 patients were affected per NO with an IQR of 6–24. The median age was 41 years (IQR: 7.5–54.5). For 1,153 patients, information on biological sex was provided (479 female; 41.5%). In 44 outbreaks (22.4%), only children were involved. Concrete information on infection or colonization was provided for 2,310 patients, which included 1,828 patients with infection (79.1%). Regarding underlying diseases, 176 articles provided data: The majority (98 articles; 55.7%) stated hematologic neoplasms whereas solid tumors and non-malignant hematologic diseases were reported in 52 (29.5%) and 26 (14.8%) of the reports, respectively. There were 49 NO reports that included patients undergoing hematopoietic stem cell transplantation.

**Fig. 1:**
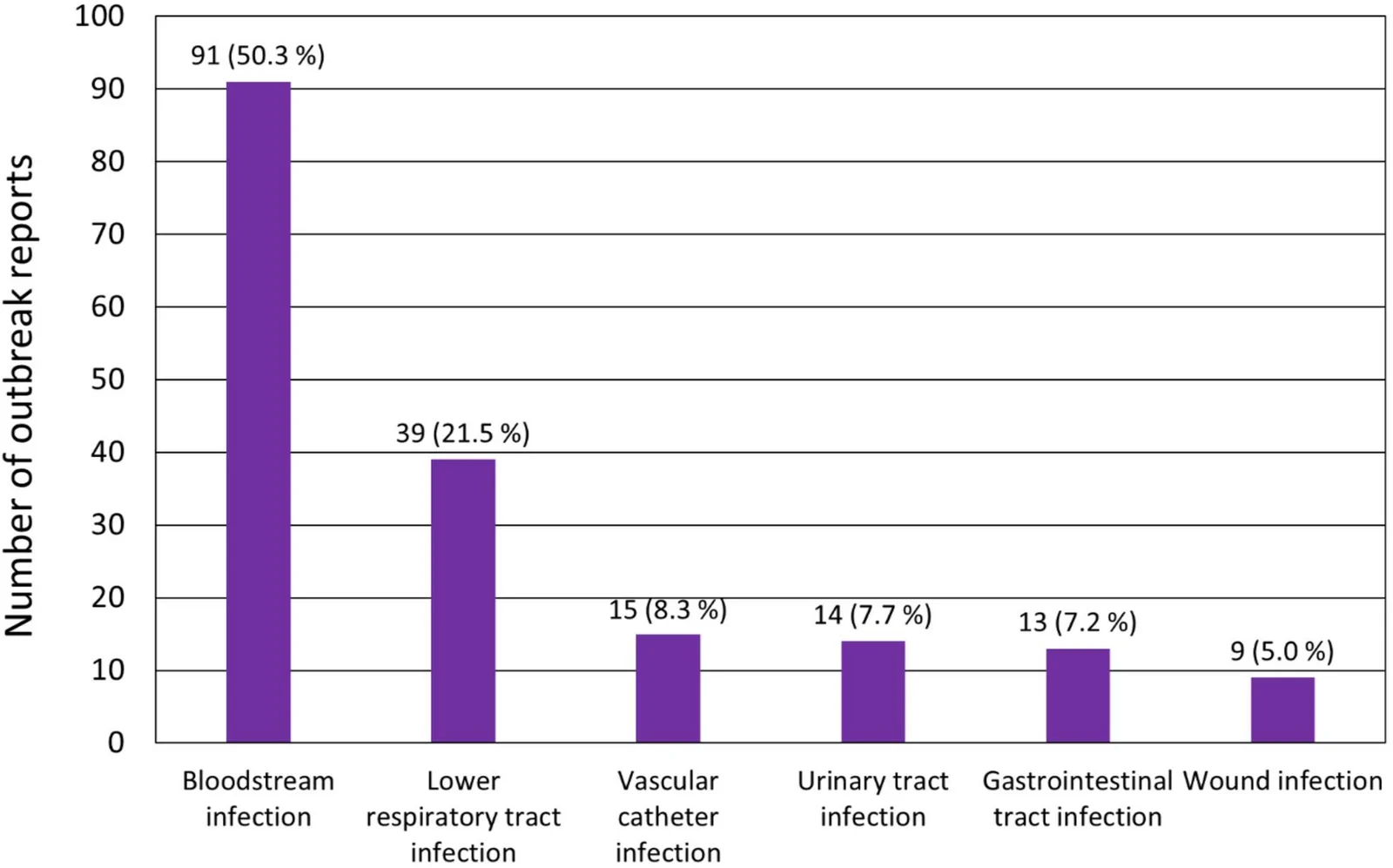
Types of infection. More than one infection can be reported per outbreak

Figure 1 shows the different types of infections reported. Bloodstream infections where the most often reported type of infection (in 91 of 196 NOs). Overall, 116 NOs gave information regarding infection-associated mortality within the NO: In 70 reports (60.3%) patients died.

### Pathogens, sources and transmission pathway

**Fig. 2:**
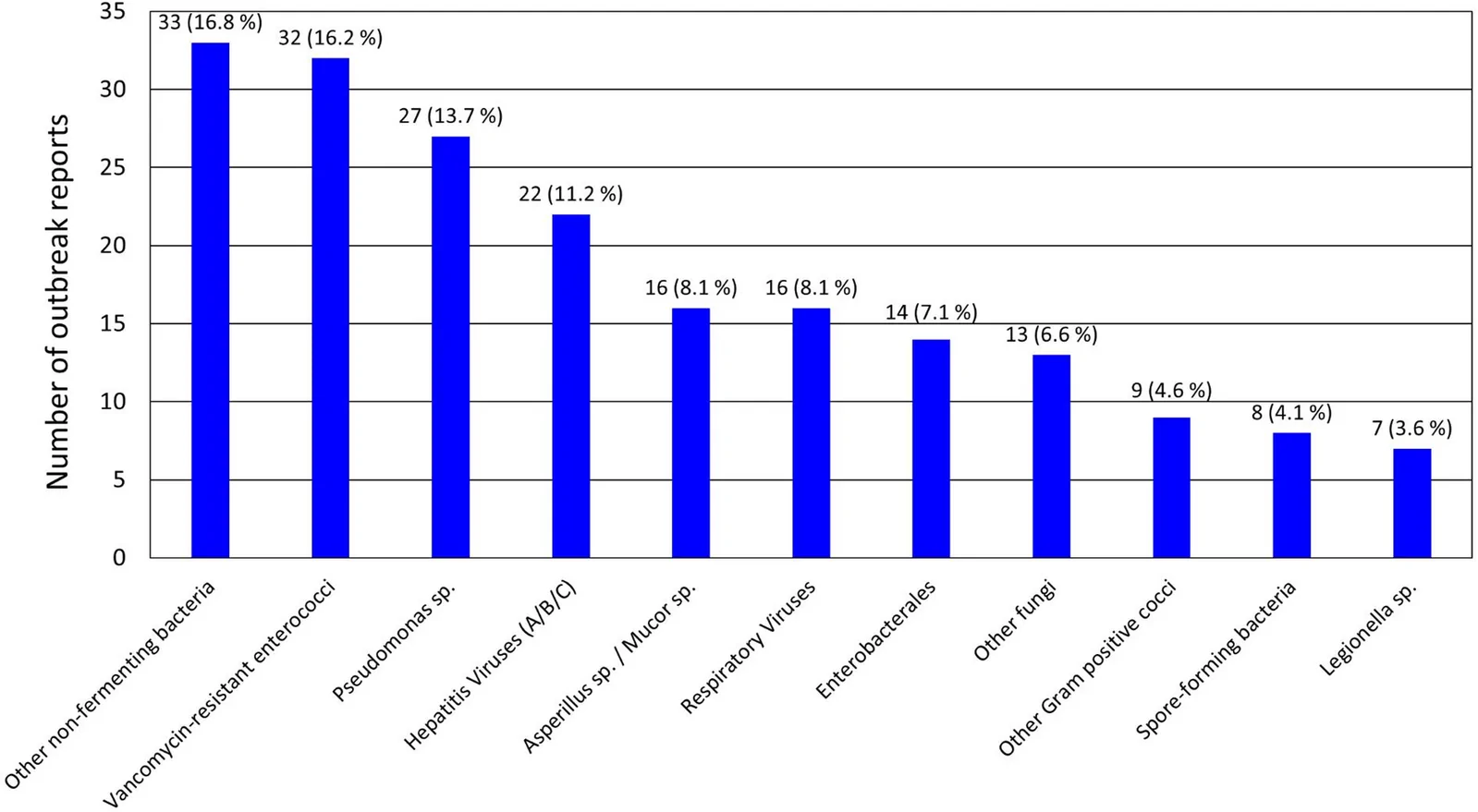
Pathogen distribution

Figure 2 provides an overview on the pathogen spectrum in the NOs. Note that *Pseudomonas sp.*, enterococci and other non-fermenting Gram-negative rods accounted for the most NOs. Multi-drug resistance was observed in 59 of the 196 NO reports (30.1%). For instance, all reported NOs caused by enterococci were caused by vancomycin-resistant strains. Despite the onset of the SARS-CoV-2 pandemic in late 2019, no NOs from H/O departments had been published by the end of our literature search period.

**Fig. 3:**
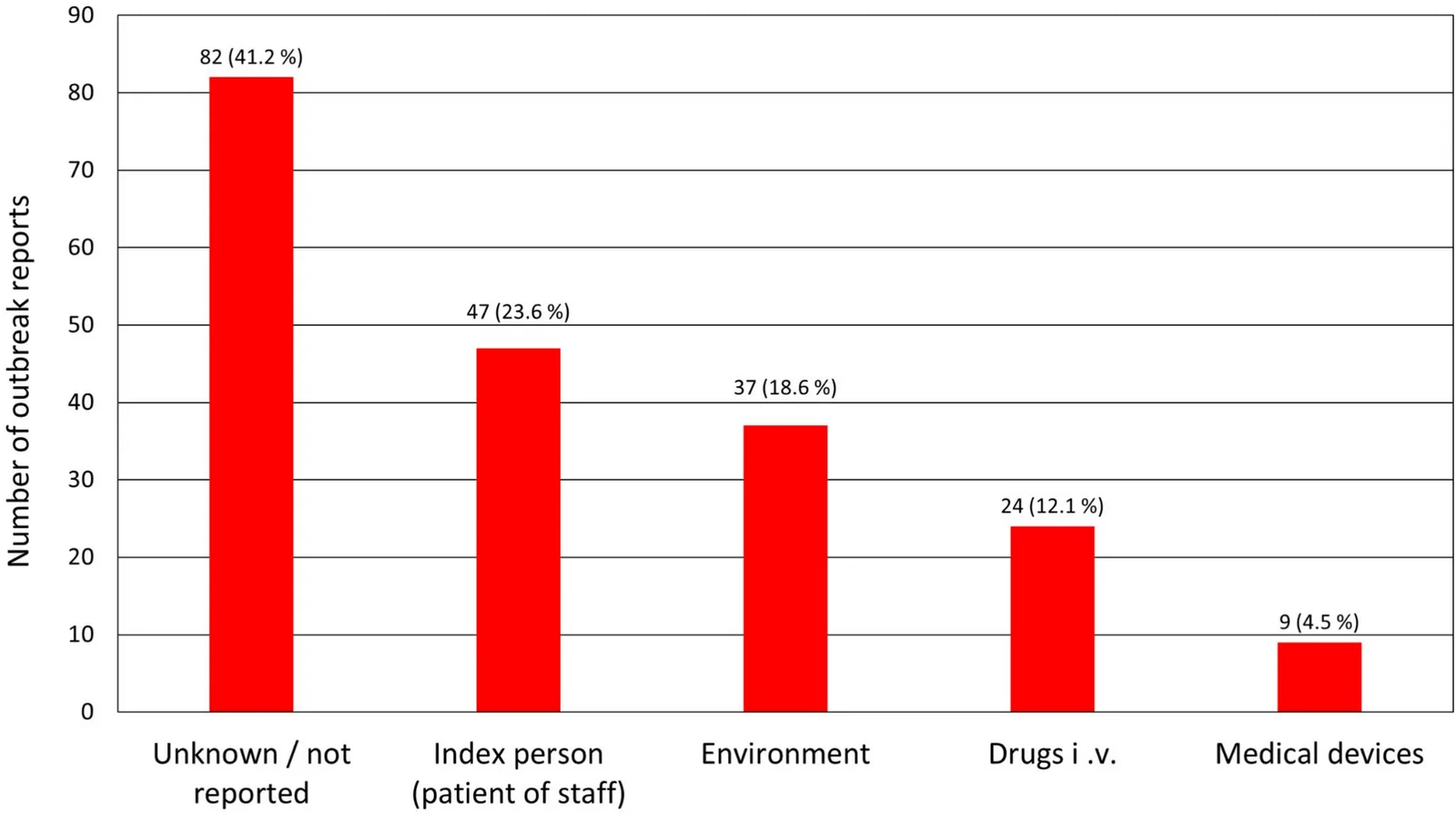
Sources of nosocomial outbreaks

Figure 3 summarizes the various sources of those pathogens. In most of the articles, a definitive source was not reported or remained unknown. There were 132 NO reports (67.3%) that provided information on the transmission pathway (multiple routes possible). Routes of pathogen transmission were mainly indirect contact, for instance via inanimate surfaces (119 NOs), or via direct person-to-person transmission (31 NOs). In 46 articles, it was specified in more detail whether a technical or human error or both made the transmission possible. Technical errors were mentioned in 19 NOs and human errors in 31 NOs.

### Infection control measures and WCHS

**Fig. 4:**
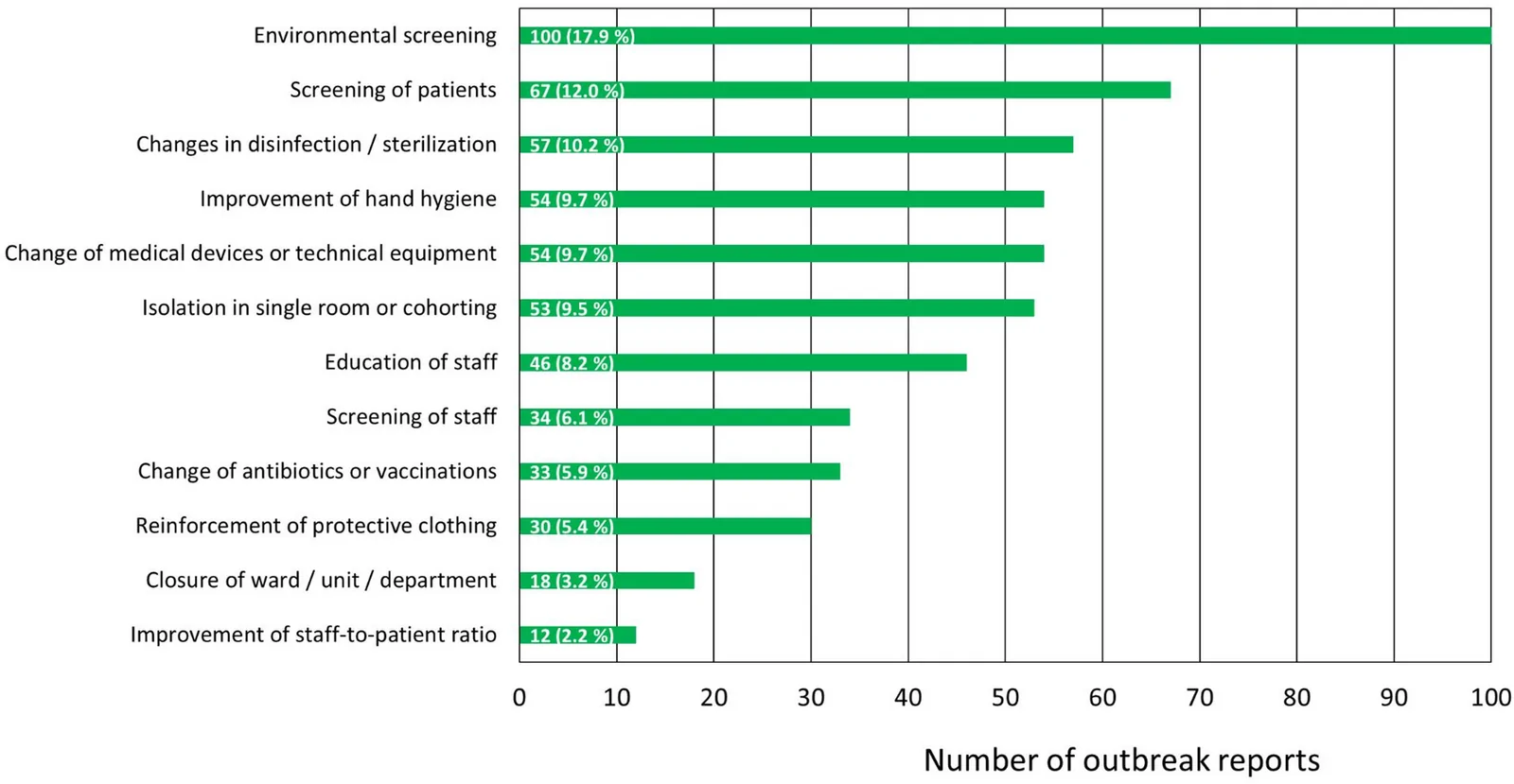
Infection control measures implemented or reinforced in nosocomial outbreaks. More than one measure can be reported per outbreak

Figure 4 shows the distribution of the IC measures, which were reported in the 196 NO reports. Note that in more than half of all NOs environmental screening of surfaces, the water supply or the air was performed. The median WCHS was 4 (IQR: 2–7). The descriptive data of all 196 NOs stratified by WCHS (low = 0, moderate > 4, high > 7) is provided as Supplemental Table B. The descriptive data of all NOs with information about mortality within the outbreak is shown in Supplemental Table C. There was no correlation between the proportion of patients who died within the NOs and the WCHS (Spearman correlation coefficient = 0.043, *p* = 0.649).

### Multivariate analyses

The results for factors independently associated with high IC efforts as expressed by the WCHS (low = 0, moderate > 4, high > 7) are shown in Table 2. Factors that were associated with a high WCHS were “Antibiotic therapy”, “Causative agent: fungi other than molds”, “Causative agent: multi-drug-resistant bacteria”, “Route of transmission: direct contact and number patients involved ≥  10 (compared to < 10 patients)”. Table 3 shows the multivariate analysis of risk factors for mortality within NOs in H/O. These were the factors “Solid tumor”. “History of use of invasive medical devices” and “Lower respiratory tract infection”.

**Table 2:** Factors independently associated with high, moderate or low infection control effort

Parameter	Odds ratio (95%CI)	*p*-value
**Factors associated with a low WCHS (= 0)**		
Source: drugs i.v.	14.39 (4.55–45.43)	< 0.0001
Route of transmission: direct or indirect contact	0.15 (0.056–0.40)	0.0002
**Factors associated with a moderate WCHS (> 4)**		
Type of infection: bloodstream infection	3.16 (1.52–6.59)	0.0022
Type of infection: lower respiratory tract infection	2.69 (1.13–6.43)	0.0261
Type of infection: vascular catheter infection	0.19 (0.04–0.80)	0.0241
Causative agent: fungi other than molds	5.69 (1.28–25.34)	0.0226
Causative agent: vancomycin-resistant enterococci	3.75 (1.45–9.70)	0.0063
Source: drugs i.v.	0.10 (0.03–0.37)	0.0006
Route of transmission: direct contact	8.56 (3.64–20.11)	< 0.0001
Staff infected	29.51 (2.42–359.40)	0.0080
Cause: human error	0.36 (0.13–0.98)	0.0458
**Factors associated with a high WCHS (> 7)**		
Antibiotic therapy	2.97 (1.05–8.38)	0.0396
Causative agent: fungi other than molds	6.26 (1.60–24.44)	0.0083
Causative agent: multi-drug-resistant bacteria	6.01 (2.56–14.09)	< 0.0001
Route of transmission: direct contact	3.42 (1.43–8.16)	0.0056
Number patients involved > = 10 (compared to < 10 patients)	2.54 (1.11–5.81)	0.0271

i.v., intravenously; WCHS, Weighted Cumulative Hygiene Score

The parameters considered in the stepwise forward variable selection were as follows: Size of the NO: Number of H/O departments (> 1), number of departments (> 1). Persons affected: personal colonized, personal infected, number of H/O patients ( > = 10), number of patients ( > = 10). Comorbidities: solid tumor, hematological-oncological disease, hematological but not oncological disease, bone marrow transplantation. Treatment: antibiotic therapy, chemotherapy, invasive devices. Groups of causative agents: bacteria, fungi, viruses. Types of causative agents: vancomycin-resistant enterococci, other Gram-positive cocci, *Pseudomonas sp*., other non-fermenting bacteria, *Enterobacterales*, *Legionella spp*., spore-forming bacteria, *Aspergillus sp.*, *Mucor sp.*, other fungi, respiratory viruses, hepatitis Viruses (A/B/C). Routes of transmission: direct contact, indirect contact. Types of infections: infected patients only (no patients with colonization), bloodstream infections, urinary tract infections, wound infections, lower respiratory tract infections, vascular catheter infections, gastrointestinal tract infections. Multi-drug resistant pathogens: multi-drug resistant bacteria in general, multi-drug resistant Gram-negative rods, vancomycin-resistant enterococci, methicillin-resistant *Staphylococcus aureus*. Sources: environment, index person (patient or staff), i.v. drugs, medical devices, source unknown. Causes: human error, technical problems of any kind

**Table 3:** Factors independently associated with mortality in nosocomial outbreaks in hematology-oncology

Parameter	Odds ratio (95%CI)	*p*-value
Solid tumor	0.187 (0.065–0.535)	0.0018
History of use of invasive medical devices	0.105 (0.034–0.325)	< 0.0001
Lower respiratory tract infection	7.95 (2.056–30.732)	0.0027

Please refer to the footnote of Table 2 for the parameters considered in the stepwise forward variable selection

## Discussion

The analysis of 196 NO reports in H/O over a 50-year period underscores their severe clinical risk, with over half of these reports documenting fatal outcomes The leading types of infections occurred in the blood stream and the lower respiratory tract. Lower respiratory tract infection was significantly associated with mortality according to the multivariate analysis. Both bacterial and viral respiratory tract infections are well-documented in the literature for their potentially severe course in H/O patients [14, 15]. Thus, appropriate protective measures, especially during an NO, are particularly important. These include but are not limited to isolation of potentially infectious patients as well as the use of personal protective equipment depending upon the type of pathogen [16–18]. Nevertheless, it is important to consider that these findings represent observed statistical correlations rather than direct causal relationships.

The majority of the analyzed NOs reports originated from Europe and North America, reflecting a certain bias. For instance, in limited-resource settings when no extra wards are available as backup, high effort measures such as closing an entire unit may just be impossible. As observed in other analyses of NOs [13], relevant contextual factors and influencing conditions were often not comprehensively reported. For instance, five NO reports did not specify the number of affected patients. However, comprehensive data are essential for a thorough understanding of NOs, including their sources and precise transmission pathways. Adherence to systematic and structured guidelines, such as the ORION statement [19], is therefore crucial and strongly recommended when reporting such events. Thus, it should be the responsibility of authors, reviewers and editors alike to ensure providing complete descriptions of NO settings [20].

The most prevalent organisms involved were *Pseudomonas sp.*, other Gram-negative non-fermenting bacteria, and enterococci. The population studied was highly vulnerable due to prolonged phases of neutropenia, mucosal barrier injury, use of medical devices, and continuous antibiotic pressure. In nearly one-third of the NO reports, the pathogens involved exhibited severe antimicrobial resistance or even multidrug resistance. This was especially evident in outbreaks caused by enterococci, as all strains were vancomycin-resistant isolates. It is worth noting that NOs caused by multidrug-resistant bacterial isolates are typically more readily identified and often prompt swift outbreak investigations. Thus, a certain publication bias regarding the presence of multidrug-resistant pathogens in the data of this study is to be expected. On the other hand, there may also be an extraordinary high consumption of antimicrobial substances in H/O especially during the phase of neutropenia [21, 22], which in turn would then increase the likelihood of selecting for pathogens with more resistant susceptibility profiles [23].

For *Pseudomonas sp.* and other Gram-negative non-fermenting bacteria, an exogenous transmission and infection pathway (e.g., from an environmental source or other patients) is particularly likely [24], although an endogenous infection may also be possible in principle, (e.g., by a leaky-gut-syndrome in asymptomatic colonized patients or in central vascular catheter infections deriving from the patient´s cutaneous flora). This emphasizes the necessity of specific protective measures against these pathogens, such as strict IC measures regarding water exposure (e.g., using sterile filters at water outlets [25, 26]), surface disinfection, but also – probably foremost – proper hand hygiene. The indirect contact transmission pathway was frequently cited in many NO reports as the mode of transmission in our study, aligning with the exogenous acquisition. For example, the inanimate environment can serve as a transmission vehicle or source if there is insufficient HEPA filtration within the air system and mold conidia get inhaled by immunosuppressed patients. More than 35 of the NO reports identified the environment as the outbreak´s source. Interestingly, in most cases, no specific source could be identified, or it was not explicitly mentioned. However, unfortunately it is rather common that the specific sources and transmission pathways remain unresolved in NOs. Currently, there are a total of 3,751 NOs filed in the Worldwide Outbreak Database, of which, the outbreak source could not be determined in 1,041. Yet, in unawareness of the exact source, only the implementation of comprehensive IC intervention bundles can effectively end the NO. Commonly implemented IC measures in our analysis included patient screening, improved cleaning/disinfection of surfaces or medical devices, enhanced adherence to hand hygiene, environmental microbiologic investigations, and isolation of affected patients. Of note, the exact screening protocols and targeted endpoints for environmental microbiologic investigations were often kept broad in primary literature, making it difficult to provide detailed insights into, for example, the frequency or specific type of environmental diagnostics performed.

In this study, the extent of the implemented outbreak control measures was classified using the previously published WCHS (weighted cumulative hygiene score; scale of 0 to 21) [13]. A high WCHS indicates the implementation of numerous IC measures or measures that are especially expensive or challenging to implement (e.g., ward closures). In the multivariable analysis with the endpoint of a moderate WCHS > 4, relevant influencing factors included lower respiratory tract infections and NOs caused by vancomycin-resistant enterococci or fungi (other than molds, e.g., opportunistic yeasts). Particularly comprehensive and intensive IC measures (WCHS > 7) were implemented when multidrug-resistant bacteria caused the NO in H/O or when > 10 patients were involved. The involvement of numerous patients may indicate a dynamic and initially uncontrolled NO, requiring increasingly comprehensive IC measures. The concern over limited treatment options in cases of infection with multidrug-resistant organisms [27] likely also plays a significant role, leading to a willingness to invest in enhanced hygiene efforts. Interestingly, no additional hygiene measures were implemented (WCHS = 0) when the outbreak´s source was a contaminated intravenous drug. In these clear monocausal NO, eliminating the point source is typically sufficient to stop the event, and further measures may often be unnecessary. The main challenge here lies in quickly identifying that very point source.

The high proportion of cases where the exact source of transmission remained unknown (or was not reported although we assume that if a source had been identified, it would have been reported) has significant implications for hospital epidemiology. In the absence of a clearly identified source, targeted interventions are not feasible. This forces healthcare facilities to adopt broad intervention bundles that address various potential environmental and procedural reservoirs simultaneously. While often effective, these non-specific bundles — encompassing intensified environmental deep-cleaning, universal screening, and rigorous staffing adjustments — are often more resource- and cost-intensive than targeted measures.

Our study has several limitations and strengths. The analysis was based on the Worldwide Outbreak Database [12], the largest global collection of NO reports. All reports underwent additional assessment before inclusion in this database. Thus, we assume that it provides a valid and robust reflection of published evidence. However, it cannot be excluded that other published NO reports to be found outside this database and, thus, were missed in this analysis. Therefore, our findings are best understood as a valuable historical map of reported outbreaks, rather than an epidemiological absolute. Additionally, it may generally be assumed that a significant proportion of actual NOs are not made publicly available through publications (so called dissemination bias or non-reporting). Moreover, the definition of what actually constitutes an “outbreak” may vary locally. In this study, we only considered events with epidemiologically related nosocomial acquisition of a pathogen by at least two patients. Potential heterogeneity may also apply to the definitions of an “infection” in contrast to a “colonization”. Furthermore, due to its nature this review can only rely on the initial clinical judgment, which is inherent limitation using this kind of approach. In addition, many included NOs lacked some relevant data points, which limits the interpretability of certain items. This applies in particular to the aspect of mortality, as the underlying outbreak reports do not always allow for a clear derivation of outbreak-attributable mortality. Furthermore, a certain baseline of all-cause mortality is inherently to be expected within the H/O patient population. As mentioned above, most of the published NO reports originated from North America and Europe, limiting the generalizability to other settings. In addition, only English-language literature was considered. These limitations must be taken into account when evaluating the evidence of the findings before application of measures in a specific local H/O setting and generalization should always be made with caution. One should also keep in mind that H/O patients considerably differ from a non-H/O patient population, for example due to prolonged phases of severe immunosuppression (neutropenia) and increased use of antimicrobial substances selecting for more resistant pathogen (prevalence of multi-drug resistance). As the data analysis period did not include the COVID-19 pandemic, no statements can be made regarding NOs with SARS-CoV-2 in H/O, which have been widely described in the literature in the last years [28, 29]. So, the interpretation and validation of the WCHS remains to be determined under these particular circumstances [30].

However, the strengths of this study include the broad data basis and the structured recording and quantification of implemented IC measures using the WCHS and the comprehensive statistical analysis for the endpoints of WCHS and mortality. To our knowledge, this is the most detailed analysis of the extent and effort in IC measures in NO occurring in H/O currently available.

In conclusion, NO in H/O severely burden patients and staff. This analysis of causes, transmission and IC interventions may provide essential guidance when managing future events as it identifies independent risk factors driving mortality and the intensity of intervention bundles. Therefore, when managing communicable lower respiratory tract infections (e.g., caused by viruses such as influenza), facemasks and patient isolation in single rooms should be implemented at the earliest possible time point. Furthermore, strict adherence to standard precautions, including appropriate surface and hand hygiene, is essential to address potential direct and indirect contact transmission. This analysis also highlights the critical importance of publishing NO reports according to standardized criteria with comprehensive and complete information. Future authors of NO reports are herewith encouraged to provide such data including data on costs or losses of revenues during an outbreak, as until now that kind of data is rather scarce.

## Supplementary Information

Below is the link to the electronic supplementary material.

Supplementary Material 1

## Data Availability

Data is provided within the manuscript or supplementary information files.
