# Supplementary material for: Lessons learned from a structured and descriptive analysis of nosocomial outbreaks in hematology-oncology
Source: BMC Infect Dis. 2026 Aug 20;26:1551. doi: 10.1186/s12879-026-14279-2 (PMC13505061; doi:10.1186/s12879-026-14279-2)
Supplement: Supplementary file 1 [file 12879_2026_14279_MOESM1_ESM.docx]

**Article Type:**	Original article

**Title:**	Lessons learned from a structured and descriptive analysis of nosocomial outbreaks in hematology-oncology

**Authors:**	Sara-Lena Posselt^1^

Claas Baier^1,3^ MD	ORCID ID: 0000-0003-3895-5446

Frank Schwab^2^ PhD	ORICD ID: 0000-0002-6666-725X

Ralf-Peter Vonberg^1^* MD	ORCID ID: 0000-0001-7706-8511

**Affiliations:**	^1^Institute for Medical Microbiology and Hospital Epidemiology, Hannover Medical School,	Hannover, Germany

^2^Institute for Hygiene and Environmental Health, Charité – University Medicine Berlin,	Berlin, Germany

^3^Infection Control Department, Agaplesion Diakonieklinikum Rotenburg, Rotenburg, Germany

***Contact:**	Prof. Ralf-Peter Vonberg

Institute for Medical Microbiology and Hospital Epidemiology

Hannover Medical School

Carl-Neuberg-Str. 1

D-30625 Hannover

Germany

Tel:	+49 (0)511 532 4431

Fax:	+49 (0)511 532 4366

eMail:	vonberg.ralf@mh-hannover.de

**SUPPLEMENTARY FILE LEGENDS**

**Supplemental Table A**

**Descriptive data of all 196 nosocomial outbreaks.**

**Supplemental Table B**

**Descriptive data of all 196 nosocomial outbreaks stratified by the Weighted Cumulative Hygiene Score (low = 0, moderate > 4, and high > 7).**

**Supplemental Table C**

**Descriptive data of 116 nosocomial outbreaks with information regarding infection-associated mortality within the outbreak.**

**Supplemental File D**

**List of all nosocomial outbreak articles includes in the systematic review.**

**Supplemental Table A**

| **Parameter** | **Category** | **Total**  **N (%) / Med (IQR)** |
| --- | --- | --- |
| **Publications of nosocomial outbreaks** | | |
| No. of publications of outbreak reports |  | 196 (100 %) |
| Publication (year) |  | 2002 (1997 – 2007) |
| Start of nosocomial outbreak (year) |  | 1997 (1993 – 2003) |
| Duration of nosocomial outbreak (weeks) |  | 18 (8 – 52) |
| **Patients and units involved in nosocomial outbreaks** | | |
| Patient’s age groups | n. s. | 60 (30.6 %) |
|  | Adults | 70 (35.7 %) |
|  | Children | 44 (22.4 %) |
|  | Adults and Children | 22 (11.2 %) |
| No. of patients |  | 11 (6 – 24) |
| No. of patients | >= 10 patients | 103 (52.6 %) |
| No. of hematology-oncology patients |  | 9 (5 – 17) |
| No. of hematology-oncology patients | >= 10 patients | 88 (44.9 %) |
| Patient’s age (median) |  | 41 (8 – 55) |
| No. of infected patients |  | 7 (5 – 13) |
| No. of units / departments |  | 1 (1 – 2) |
| No. of units / departments | n. s. | 52 (26.5 %) |
|  | 1 unit / department | 93 (47.4 %) |
|  | > 1 unit / department | 51 (26.0 %) |
| No. of oncological units / departments |  | 1 (1 – 1) |
| No. of oncological units / departments | n. s. | 53 (27.0 %) |
|  | 1 unit / department | 118 (60.2 %) |
|  | > 1 unit / department | 25 (12.8 %) |
| **Nosocomial outbreaks in which patients with comorbidities and treatments were involved** | | |
| Solid tumor |  | 52 (26.5 %) |
| Hematological-oncological |  | 98 (50.0 %) |
| Hematological and not oncological |  | 26 (13.3 %) |
| Bone marrow transplantation |  | 49 (25.0 %) |
| Antibiotic therapy |  | 22 (11.2 %) |
| Chemotherapy |  | 42 (21.4 %) |
| Invasive devices |  | 45 (23.0 %) |
| **Groups of causative agents in nosocomial outbreaks** | | |
| Bacteria |  | 119 (60.7 %) |
| Fungi |  | 32 (16.3 %) |
| Viruses |  | 42 (21.4 %) |
| **Types of causative agents in nosocomial outbreaks** | | |
| Vancomycin-resistant enterococci |  | 34 (17.3 %) |
| Other Gram-positive cocci |  | 9 (4.6 %) |
| Other non-fermenting bacteria |  | 33 (16.8 %) |
| *Pseudomonas sp.* |  | 27 (13.8 %) |
| *Enterobacterales* |  | 14 (7.1 %) |
| *Legionella sp.* |  | 7 (3.6 %) |
| Spore-forming bacteria |  | 8 (4.1 %) |
| *Aspergillus sp.* / *Mucor sp.* |  | 16 (8.2 %) |
| Other fungi |  | 13 (6.6 %) |
| Respiratory viruses |  | 16 (8.2 %) |
| Hepatitis viruses (A/B/C) |  | 22 (11.2%) |
| **Routes of transmission in nosocomial outbreaks** | | |
| Direct contact |  | 31 (15.8 %) |
| Indirect contact |  | 119 (60.7 %) |
| **Types of infections in nosocomial outbreaks** | | |
| Infected patients only (no patients with colonizations) |  | 121 (75.6 %) |
| Bloodstream infection |  | 91 (46.4 %) |
| Urinary tract infection |  | 14 (7.1 %) |
| Wound infection |  | 9 (4.6 %) |
| Lower respiratory tract infection |  | 39 (19.9 %) |
| Vascular catheter infection |  | 15 (7.7 %) |
| Gastrointestinal tract infection |  | 13 (6.6 %) |
| **Multi-drug resistant pathogens involved in nosocomial outbreaks** | | |
| Multi-drug resistant bacteria |  | 59 (30.1 %) |
| Multi-drug resistant Gram-negative rods |  | 20 (10.2 %) |
| Vancomycin-resistant enterococci |  | 34 (17.3 %) |
| Methicillin-resistant *Staphylococcus aureus* |  | 5 (2.6 %) |
| **Sources of nosocomial outbreaks** | | |
| Environment |  | 37 (18.9 %) |
| Index person (patient or staff) |  | 47 (24.0 %) |
| Drugs i.v. |  | 24 (12.2 %) |
| Medical devices |  | 9 (4.6 %) |
| Unknown or not reported |  | 82 (41.8 %) |
| **Causes of nosocomial outbreaks** | | |
| Human error |  | 31 (15.8 %) |
| Technical problem |  | 19 (9.7 %) |
| **Infection control measures in nosocomial outbreaks** | | |
| Screening of staff |  | 34 (17.3 %) |
| Closure of the ward or department |  | 18 (9.2 %) |
| Improvement of staff-to-patient ratio or other changes in staffing |  | 12 (6.1 %) |
| Environmental screening |  | 100 (51.0 %) |
| Surfaces |  | 88 (44.9 %) |
| Air |  | 20 (10.2 %) |
| Water |  | 34 (17.3 %) |
| Changes in disinfection and/or sterilization procedures |  | 57 (29.1 %) |
| Changes in handling of technical equipment |  | 54 (27.6 %) |
| Screening of patients |  | 67 (34.2 %) |
| Isolation (single room or cohort) |  | 53 (27.0 %) |
| Change of antibiotics or vaccinations |  | 33 (16.8 %) |
| Change of antibiotic therapy |  | 18 (9.2 %) |
| Change of prophylaxis |  | 11 (5.6 %) |
| Vaccination |  | 5 (2.6 %) |
| Improvement of hand hygiene |  | 54 (27.6 %) |
| Reinforcement of protective clothing |  | 30 (15.3 %) |
| Training and education of staff |  | 46 (23.5 %) |
| **Weighted cumulative hygiene score (WCHS)** | | |
| WCHS (points) |  | 4 (2 – 7) |
| WCHS | = 0 points | 35 (17.9 %) |
| WCHS | > 4 points | 93 (47.4 %) |
| WCHS | > 7 points | 44 (22.4 %) |
| **No. of patients with information about mortality in nosocomial outbreaks** | | |
| Patients who died during the outbreak | no | 46 (39.7 %) |
|  | yes | 70 (60.3 %) |
| No. patients who survived |  | 7 (4 – 14) |
| No. patients who died |  | 1 (0 – 4) |

No., number; Med, Median; IQR, interquartile range; n. s., not specified

**Supplemental Table B**

| **Parameter** | **Category** | **low WCHS** | | **% of nosocomial outbreaks with**  **WCHS = 0** | **p-value** | **moderate WCHS** | | **% of nosocomial outbreaks with**  **WCHS > 4** | **p-value** | **high WCHS** | | **% of nosocomial outbreaks with**  **WCHS > 7** | **p-value** |
| --- | --- | --- | --- | --- | --- | --- | --- | --- | --- | --- | --- | --- | --- |
|  |  | **>0** | **=0** |  |  | **<=4** | **>4** |  |  | **<=7** | **>7** |  |  |
|  |  | **N (%) / Med (IQR)** | |  |  | **N (%) / Med (IQR)** | |  |  | **N (%) / Med (IQR)** | |  |  |
| **Publications of nosocomial outbreaks** | | | | | | | | | | | | | |
| No. of publications of nosocomial outbreaks |  | 161 (100 %) | 35 (100 %) | 17.9 |  | 103 (100 %) | 93 (100 %) | 47.4 |  | 152 (100 %) | 44 (100 %) | 22.4 |  |
| Publication (year) |  | 2002  (1996 – 2007) | 2002  (1997 – 2006) |  | 0.797 | 2002  (1995 – 2007) | 2002  (1998 – 2007) |  | 0.105 | 2002  (1996 – 2007) | 2002  (1999 – 2006) |  | 0.629 |
| Start of nosocomial outbreak (year) |  | 1997  (1992 – 2004) | 1998  (1994 – 2002) |  | 0.994 | 1997  (1991 – 2002) | 1998  (1994 – 2004) |  | 0.064 | 1997  (1992 – 2003) | 1998  (1995 – 2004) |  | 0.335 |
| Duration of nosocomial outbreak (weeks) |  | 15 (7 – 48) | 35 (14 – 70) |  | 0.068 | 21 (8 – 69) | 17 (8 – 48) |  | 0.530 | 17 (6 – 65) | 20 (9 – 43) |  | 0.690 |
| **Patients and units involved in nosocomial outbreaks** | | | | | | | | | | | | | |
| Patient´s age groups | n. s. | 49 (30.4 %) | 11 (31.4 %) | 18.3 | 0.098 | 32 (31.1 %) | 28 (30.1 %) | 46.7 | 0.133 | 44 (28.9 %) | 16 (36.4 %) | 26.7 | 0.096 |
|  | Adults | 60 (37.3 %) | 10 (28.6 %) | 14.3 |  | 31 (30.1 %) | 39 (41.9 %) | 55.7 |  | 50 (32.9 %) | 20 (45.5 %) | 28.6 |  |
|  | Children | 38 (23.6 %) | 6 (17.1 %) | 13.6 |  | 24 (23.3 %) | 20 (21.5 %) | 45.5 |  | 38 (25.0 %) | 6 (13.6 %) | 13.6 |  |
|  | Adults and children | 14 (8.7 %) | 8 (22.9 %) | 36.4 |  | 16 (15.5 %) | 6 (6.5 %) | 27.3 |  | 20 (13.2 %) | 2 (4.5 %) | 9.1 |  |
| No. of patients |  | 11 (6 – 23) | 11 (7 – 30) |  | 0.608 | 10 (6 – 24) | 12 (7 – 24) |  | 0.248 | 10 (6 – 24) | 16 (8 – 23) |  | 0.164 |
| No. of patients | >= 10 patients | 85 (52.8 %) | 18 (51.4 %) | 17.5 | 0.883 | 50 (48.5 %) | 53 (57.0 %) | 51.5 | 0.237 | 74 (48.7 %) | 29 (65.9 %) | 28.2 | 0.044 |
| No. of hematology-oncology patients |  | 8 (5 – 17) | 10 (4 – 25) |  | 0.750 | 9 (5 – 16) | 8 (6 – 19) |  | 0.509 | 8 (5 – 15) | 11 (6 – 21) |  | 0.212 |
| No. of hematology-oncology patients | >= 10 patients | 72 (44.7 %) | 16 (45.7 %) | 18.2 | 0.915 | 47 (45.6 %) | 41 (44.1 %) | 46.6 | 0.828 | 66 (43.4 %) | 22 (50.0 %) | 25.0 | 0.440 |
| Patient’s age (median) |  | 45 (7 – 55) | 36.5 (11-52) |  | 0.570 | 38 (7 – 54) | 45.5 (8 – 58) |  | 0.367 | 39 (7 – 57) | 45 (33 – 52) |  | 0.859 |
| No. of infected patients |  | 7 (5 – 12) | 9 (4 – 24) |  | 0.253 | 8 (5 – 14) | 6 (4 – 11) |  | 0.193 | 8 (5 – 14) | 6 (4 – 10) |  | 0.082 |
| No. of units / departments |  | 1 (1 – 2) | 2 (1 – 2) |  | 0.154 | 1 (1 – 2) | 1 (1 – 2) |  | 0.991 | 1 (1 – 2) | 1 (1 – 2) |  | 0.614 |
| No. of units / departments | n. s. | 39 (24.2 %) | 13 (37.1 %) | 25.0 | 0.101 | 33 (32.0 %) | 19 (20.4 %) | 36.5 | 0.184 | 45 (29.6 %) | 7 (15.9 %) | 13.5 | 0.132 |
|  | 1 unit / department | 82 (50.9 %) | 11 (31.4 %) | 11.8 |  | 45 (43.7 %) | 48 (51.6 %) | 51.6 |  | 67 (44.1 %) | 26 (59.1 %) | 28.0 |  |
|  | > 1 unit / department | 40 (24.8 %) | 11 (31.4 %) | 21.6 |  | 25 (24.3 %) | 26 (28.0 %) | 51.0 |  | 40 (26.3 %) | 11 (25.0 %) | 21.6 |  |
| No. of oncological units / departments |  | 1 (1 – 1) | 1 (1 – 1) |  | 0.849 | 1 (1 – 1) | 1 (1 – 1) |  | 0.980 | 1 (1 – 1) | 1 (1 – 1) |  | 0.264 |
| No. of oncological units / departments | n. s. | 39 (24.2 %) | 14 (40.0 %) | 26.4 | 0.160 | 34 (33.0 %) | 19 (20.4 %) | 35.8 | 0.141 | 46 (30.3 %) | 7 (15.9 %) | 13.2 | 0.072 |
|  | 1 unit | 101 (62.7 %) | 17 (48.6 %) | 14.4 |  | 57 (55.3 %) | 61 (65.6 %) | 51.7 |  | 85 (55.9 %) | 33 (75.0 %) | 28.0 |  |
|  | > 1 unit | 21 (13.0 %) | 4 (11.4 %) | 16.0 |  | 12 (11.7 %) | 13 (14.0 %) | 52.0 |  | 21 (13.8 %) | 4 (9.1 %) | 16.0 |  |
| **Nosocomial outbreaks in which patients with comorbidities and treatments were involved** | | | | | | | | | | | | | |
| Solid tumor |  | 45 (28.0 %) | 7 (20.0 %) | 13.5 | 0.334 | 27 (26.2 %) | 25 (26.9 %) | 48.1 | 0.916 | 42 (27.6 %) | 10 (22.7 %) | 19.2 | 0.516 |
| Hematological-oncological |  | 87 (54.0 %) | 11 (31.4 %) | 11.2 | 0.015 | 48 (46.6 %) | 50 (53.8 %) | 51.0 | 0.317 | 74 (48.7 %) | 24 (54.5 %) | 24.5 | 0.493 |
| Hematological and not oncological |  | 20 (12.4 %) | 6 (17.1 %) | 23.1 | 0.456 | 14 (13.6 %) | 12 (12.9 %) | 46.2 | 0.887 | 22 (14.5 %) | 4 (9.1 %) | 15.4 | 0.354 |
| Bone marrow transplantation |  | 44 (27.3 %) | 5 (14.3 %) | 10.2 | 0.106 | 21 (20.4 %) | 28 (30.1 %) | 57.1 | 0.117 | 38 (25.0 %) | 11 (25.0 %) | 22.4 | 1.000 |
| Antibiotic therapy |  | 21 (13.0 %) | 1 (2.9 %) | 4.5 | 0.084 | 7 (6.8 %) | 15 (16.1 %) | 68.2 | 0.039 | 12 (7.9 %) | 10 (22.7 %) | 45.5 | 0.006 |
| Chemotherapy |  | 38 (23.6 %) | 4 (11.4 %) | 9.5 | 0.112 | 21 (20.4 %) | 21 (22.6 %) | 50.0 | 0.709 | 32 (21.1 %) | 10 (22.7 %) | 23.8 | 0.812 |
| Invasive devices |  | 37 (23.0 %) | 8 (22.9 %) | 17.8 | 0.987 | 30 (29.1 %) | 15 (16.1 %) | 33.3 | 0.031 | 39 (25.7 %) | 6 (13.6 %) | 13.3 | 0.095 |
| **Groups of causative agents in nosocomial outbreaks** | | | | | | | | | | | | | |
| Bacteria |  | 101 (62.7 %) | 18 (51.4 %) | 15.1 | 0.215 | 60 (58.3 %) | 59 (63.4 %) | 49.6 | 0.458 | 88 (57.9 %) | 31 (70.5 %) | 26.1 | 0.133 |
| Fungi |  | 27 (16.8 %) | 5 (14.3 %) | 15.6 | 0.719 | 14 (13.6 %) | 18 (19.4 %) | 56.3 | 0.276 | 24 (15.8 %) | 8 (18.2 %) | 25.0 | 0.705 |
| Viruses |  | 30 (18.6 %) | 12 (34.3 %) | 28.6 | 0.041 | 27 (26.2 %) | 15 (16.1 %) | 35.7 | 0.086 | 37 (24.3 %) | 5 (11.4 %) | 11.9 | 0.065 |
| **Types of causative agents in nosocomial outbreaks** | | | | | | | | | | | | | |
| Vancomycin-resistant enterococci |  | 29 (18.0 %) | 5 (14.3 %) | 14.7 | 0.598 | 14 (13.6 %) | 20 (21.5 %) | 58.8 | 0.144 | 19 (12.5 %) | 15 (34.1 %) | 44.1 | <0.001 |
| Other Gram-positive cocci |  | 9 (5.6 %) | 0 (0.0 %) | 0.0 | 0.152 | 4 (3.9 %) | 5 (5.4 %) | 55.6 | 0.618 | 6 (3.9 %) | 3 (6.8 %) | 33.3 | 0.423 |
| Other non-fermenting bacteria |  | 28 (17.4 %) | 5 (14.3 %) | 15.2 | 0.656 | 21 (20.4 %) | 12 (12.9 %) | 36.4 | 0.162 | 28 (18.4 %) | 5 (11.4 %) | 15.2 | 0.271 |
| *Pseudomonas sp.* |  | 24 (14.9 %) | 3 (8.6 %) | 11.1 | 0.324 | 16 (15.5 %) | 11 (11.8 %) | 40.7 | 0.452 | 23 (15.1 %) | 4 (9.1 %) | 14.8 | 0.306 |
| *Enterobacterales* |  | 11 (6.8 %) | 3 (8.6 %) | 21.4 | 0.717 | 5 (4.9 %) | 9 (9.7 %) | 64.3 | 0.190 | 10 (6.6 %) | 4 (9.1 %) | 28.6 | 0.569 |
| *Legionella sp.* |  | 7 (4.3 %) | 0 (0.0 %) | 0.0 | 0.209 | 3 (2.9 %) | 4 (4.3 %) | 57.1 | 0.601 | 6 (3.9 %) | 1 (2.3 %) | 14.3 | 0.598 |
| Spore-forming bacteria |  | 6 (3.7 %) | 2 (5.7 %) | 25.0 | 0.590 | 6 (5.8 %) | 2 (2.2 %) | 25.0 | 0.194 | 7 (4.6 %) | 1 (2.3 %) | 12.5 | 0.491 |
| *Aspergillus sp.* / *Mucor sp.* |  | 15 (9.3 %) | 1 (2.9 %) | 6.3 | 0.206 | 6 (5.8 %) | 10 (10.8 %) | 62.5 | 0.208 | 11 (7.2 %) | 5 (11.4 %) | 31.3 | 0.379 |
| Other fungi |  | 13 (8.1 %) | 0 (0.0 %) | 0.0 | 0.082 | 3 (2.9 %) | 10 (10.8 %) | 76.9 | 0.028 | 8 (5.3 %) | 5 (11.4 %) | 38.5 | 0.152 |
| Respiratory viruses |  | 14 (8.7 %) | 2 (5.7 %) | 12.5 | 0.559 | 8 (7.8 %) | 8 (8.6 %) | 50.0 | 0.831 | 13 (8.6 %) | 3 (6.8 %) | 18.8 | 0.711 |
| Hepatitis viruses (A/B/C) |  | 12 (7.5 %) | 10 (28.6 %) | 45.5 | <0.001 | 16 (15.5 %) | 6 (6.5 %) | 27.3 | 0.044 | 20 (13.2 %) | 2 (4.5 %) | 9.1 | 0.111 |
| **Routes of transmission in nosocomial outbreaks** | | | | | | | | | | | | | |
| Direct contact |  | 30 (18.6 %) | 1 (2.9 %) | 3.2 | 0.020 | 14 (13.6 %) | 17 (18.3 %) | 54.8 | 0.369 | 22 (14.5 %) | 9 (20.5 %) | 29.0 | 0.338 |
| Indirect contact |  | 103 (64.0 %) | 16 (45.7 %) | 13.4 | 0.045 | 53 (51.5 %) | 66 (71.0 %) | 55.5 | 0.005 | 88 (57.9 %) | 31 (70.5 %) | 26.1 | 0.133 |
| **Types of infections in nosocomial outbreaks** | | | | | | | | | | | | | |
| Infected patients only  (no patients with colonizations) |  | 99 (73.9 %) | 22 (84.6 %) | 18.2 | 0.243 | 67 (82.7 %) | 54 (68.4 %) | 44.6 | 0.034 | 100 (80.6 %) | 21 (58.3 %) | 17.4 | 0.006 |
| Bloodstream infection |  | 77 (47.8 %) | 14 (40.0 %) | 15.4 | 0.400 | 41 (39.8 %) | 50 (53.8 %) | 54.9 | 0.050 | 68 (44.7 %) | 23 (52.3 %) | 25.3 | 0.377 |
| Urinary tract infection |  | 13 (8.1 %) | 1 (2.9 %) | 7.1 | 0.277 | 4 (3.9 %) | 10 (10.8 %) | 71.4 | 0.062 | 8 (5.3 %) | 6 (13.6 %) | 42.9 | 0.058 |
| Wound infection |  | 9 (5.6 %) | 0 (0.0 %) | 0.0 | 0.152 | 2 (1.9 %) | 7 (7.5 %) | 77.8 | 0.062 | 5 (3.3 %) | 4 (9.1 %) | 44.4 | 0.105 |
| Lower respiratory tract infection |  | 36 (22.4 %) | 3 (8.6 %) | 7.7 | 0.064 | 15 (14.6 %) | 24 (25.8 %) | 61.5 | 0.049 | 29 (19.1 %) | 10 (22.7 %) | 25.6 | 0.593 |
| Vascular catheter infection |  | 13 (8.1 %) | 2 (5.7 %) | 13.3 | 0.634 | 11 (10.7 %) | 4 (4.3 %) | 26.7 | 0.093 | 12 (7.9 %) | 3 (6.8 %) | 20.0 | 0.813 |
| Gastrointestinal tract infection |  | 11 (6.8 %) | 2 (5.7 %) | 15.4 | 0.810 | 6 (5.8 %) | 7 (7.5 %) | 53.8 | 0.633 | 11 (7.2 %) | 2 (4.5 %) | 15.4 | 0.528 |
| **Multi-drug resistant pathogens involved in nosocomial outbreaks** | | | | | | | | | | | | | |
| Multi-drug resistant bacteria |  | 48 (29.8 %) | 11 (31.4 %) | 18.6 | 0.850 | 27 (26.2 %) | 32 (34.4 %) | 54.2 | 0.212 | 35 (23.0 %) | 24 (54.5 %) | 40.7 | <0.001 |
| Multi-drug resistant Gram-negative rods |  | 14 (8.7 %) | 6 (17.1 %) | 30.0 | 0.135 | 11 (10.7 %) | 9 (9.7 %) | 45.0 | 0.817 | 14 (9.2 %) | 6 (13.6 %) | 30.0 | 0.393 |
| Vancomycin-resistant enterococci |  | 29 (18.0 %) | 5 (14.3 %) | 14.7 | 0.598 | 14 (13.6 %) | 20 (21.5 %) | 58.8 | 0.144 | 19 (12.5 %) | 15 (34.1 %) | 44.1 | <0.001 |
| Methicillin-resistant *Staphylococcus aureus* |  | 5 (3.1 %) | 0 (0.0 %) | 0.0 | 0.291 | 2 (1.9 %) | 3 (3.2 %) | 60.0 | 0.569 | 2 (1.3 %) | 3 (6.8 %) | 60.0 | 0.041 |
| **Sources of nosocomial outbreaks** | | | | | | | | | | | | | |
| Environment |  | 37 (23.0 %) | 0 (0.0 %) | 0.0 | 0.002 | 11 (10.7 %) | 26 (28.0 %) | 70.3 | 0.002 | 28 (18. 4%) | 9 (20.5 %) | 24.3 | 0.761 |
| Index person (patient or staff) |  | 41 (25.5 %) | 6 (17.1 %) | 12.8 | 0.296 | 21 (20.4 %) | 26 (28.0 %) | 55.3 | 0.215 | 32 (21.1 %) | 15 (34.1 %) | 31.9 | 0.074 |
| Drugs i.v. |  | 13 (8.1 %) | 11 (31.4 %) | 45.8 | <0.001 | 20 (19.4 %) | 4 (4.3 %) | 16.7 | 0.001 | 22 (14.5 %) | 2 (4.5 %) | 8.3 | 0.077 |
| Medical devices |  | 9 (5.6 %) | 0 (0.0 %) | 0.0 | 0.152 | 4 (3.9 %) | 5 (5.4 %) | 55.6 | 0.618 | 8 (5.3 %) | 1 (2.3 %) | 11.1 | 0.404 |
| Unknown |  | 63 (39.1 %) | 19 (54.3 %) | 23.2 | 0.099 | 49 (47.6 %) | 33 (35.5 %) | 40.2 | 0.087 | 64 (42.1 %) | 18 (40.9 %) | 22.0 | 0.887 |
| **Causes of nosocomial outbreaks** | | | | | | | | | | | | | |
| Human error |  | 24 (14.9 %) | 7 (20.0 %) | 22.6 | 0.454 | 17 (16.5 %) | 14 (15.1 %) | 45.2 | 0.781 | 24 (15.8 %) | 7 (15.9 %) | 22.6 | 0.985 |
| Technical problem |  | 17 (10.6 %) | 2 (5.7 %) | 10.5 | 0.380 | 8 (7.8 %) | 11 (11.8 %) | 57.9 | 0.337 | 15 (9.9 %) | 4 (9.1 %) | 21.1 | 0.878 |
| **Infection control measures in nosocomial outbreaks** | | | | | | | | | | | | | |
| Screening of staff |  | 34 (21.1 %) | 0 (0.0 %) | 0.0 | 0.003 | 6 (5.8 %) | 28 (30.1 %) | 82.4 | <0.001 | 17 (11.2 %) | 17 (38.6 %) | 50.0 | <0.001 |
| Closure of the ward or department |  | 18 (11.2 %) | 0 (0.0 %) | 0.0 | 0.038 | 2 (1.9 %) | 16 (17.2 %) | 88.9 | <0.001 | 6 (3.9 %) | 12 (27.3 %) | 66.7 | <0.001 |
| Improvement of staff-to-patient ratio or other changes in staffing |  | 12 (7.5 %) | 0 (0.0 %) | 0.0 | 0.096 | 0 (0.0 %) | 12 (12.9 %) | 100.0 | <0.001 | 1 (0.7 %) | 11 (25.0 %) | 91.7 | <0.001 |
| Any environmental screening |  | 100 (62.1 %) | 0 (0.0 %) | 0.0 | <0.001 | 32 (31.1 %) | 68 (73.1 %) | 68.0 | <0.001 | 63 (41.4 %) | 37 (84.1 %) | 37.0 | <0.001 |
| Surfaces |  | 88 (54.7 %) | 0 (0.0 %) | 0.0 | <0.001 | 28 (27.2 %) | 60 (64.5 %) | 68.2 | <0.001 | 55 (36.2 %) | 33 (75.0 %) | 37.5 | <0.001 |
| Air |  | 20 (12.4 %) | 0 (0.0 %) | 0.0 | 0.028 | 4 (3.9 %) | 16 (17.2 %) | 80.0 | 0.002 | 13 (8.6 %) | 7 (15.9 %) | 35.0 | 0.156 |
| Water |  | 34 (21.1 %) | 0 (0.0 %) | 0.0 | 0.003 | 15 (14.6 %) | 19 (20.4 %) | 55.9 | 0.279 | 27 (17.8 %) | 7 (15.9 %) | 20.6 | 0.775 |
| Changes in disinfection and/or sterilization procedures |  | 57 (35.4 %) | 0 (0.0 %) | 0.0 | <0.001 | 5 (4.9 %) | 52 (55.9 %) | 91.2 | <0.001 | 29 (19.1 %) | 28 (63.6 %) | 49.1 | <0.001 |
| Changes in handling of technical equipment |  | 54 (33.5 %) | 0 (0.0 %) | 0.0 | <0.001 | 8 (7.8 %) | 46 (49.5 %) | 85.2 | <0.001 | 31 (20.4 %) | 23 (52.3 %) | 42.6 | <0.001 |
| Screening of patients |  | 67 (41.6 %) | 0 (0.0 %) | 0.0 | <0.001 | 18 (17.5 %) | 49 (52.7 %) | 73.1 | <0.001 | 38 (25.0 %) | 29 (65.9 %) | 43.3 | <0.001 |
| Isolation  (single room or cohort) |  | 53 (32.9 %) | 0 (0.0 %) | 0.0 | <0.001 | 15 (14.6 %) | 38 (40.9 %) | 71.7 | <0.001 | 28 (18.4 %) | 25 (56.8 %) | 47.2 | <0.001 |
| Change of antibiotics or vaccinations |  | 33 (20.5 %) | 0 (0.0 %) | 0.0 | 0.003 | 6 (5.8 %) | 27 (29.0 %) | 81.8 | <0.001 | 14 (9.2 %) | 19 (43.2 %) | 57.6 | <0.001 |
| Change of antibiotic therapy |  | 18 (11.2 %) | 0 (0.0 %) | 0.0 | 0.038 | 1 (1.0 %) | 17 (18.3 %) | 94.4 | <0.001 | 4 (2.6 %) | 14 (31.8 %) | 77.8 | <0.001 |
| Change of prophylaxis |  | 11 (6.8 %) | 0 (0.0 %) | 0.0 | 0.111 | 4 (3.9 %) | 7 (7.5 %) | 63.6 | 0.268 | 8 (5.3 %) | 3 (6.8 %) | 27.3 | 0.693 |
| Vaccination |  | 5 (3.1 %) | 0 (0.0 %) | 0.0 | 0.291 | 2 (1.9 %) | 3 (3.2 %) | 60.0 | 0.569 | 3 (2.0 %) | 2 (4.5 %) | 40.0 | 0.341 |
| Improvement of hand hygiene |  | 54 (33.5 %) | 0 (0.0 %) | 0.0 | <0.001 | 9 (8.7 %) | 45 (48.4 %) | 83.3 | <0.001 | 26 (17.1 %) | 28 (63.6 %) | 51.9 | <0.001 |
| Reinforcement of protective clothing |  | 30 (18.6 %) | 0 (0.0 %) | 0.0 | 0.006 | 3 (2.9 %) | 27 (29.0 %) | 90.0 | <0.001 | 14 (9.2 %) | 16 (36.4 %) | 53.3 | <0.001 |
| Training and education of staff |  | 46 (28.6 %) | 0 (0.0 %) | 0.0 | <0.001 | 5 (4.9 %) | 41 (44.1 %) | 89.1 | <0.001 | 21 (13.8 %) | 25 (56.8 %) | 54.3 | <0.001 |
| **No. of patients with information about mortality in nosocomial outbreaks** | | | | | | | | | | | | | |
| Patients who died during the outbreak |  | 59 (61.5 %) | 11 (55 %) | 15.7 | 0.591 | 34 (57.6 %) | 36 (63.2 %) | 51.4 | 0.543 | 53 (58.9 %) | 17 (65.4 %) | 24.3 | 0.551 |
| No. of patients who survived |  | 7 (4 – 14) | 4 (2 – 14) |  | 0.250 | 7 (2 – 13) | 7 (4 – 15) |  | 0.191 | 6 (3 – 13) | 8 (4 – 14) |  | 0.350 |
| No. of patients who died |  | 1 (0 – 3) | 2 (0 – 5) |  | 0.529 | 1 (0 – 3) | 1 (0 – 4) |  | 0.567 | 1 (0 – 3) | 2 (0 – 4) |  | 0.383 |

No., number; Med, Median; IQR, interquartile range; n. s., not specified; WCHS, Weighted Cumulative Hygiene Score

**Supplemental Table C**

| **Parameter** | **Category** | **patients who died during the nosocomial outbreak** | | **% of nosocomial outbreaks in which patients died** | **p-value** |
| --- | --- | --- | --- | --- | --- |
|  |  | **no** | **yes** |  |  |
|  |  | **N (%) / Med (IQR)** | |  |  |
| **Publications of nosocomial outbreaks** | | | | | |
| No. of publications of outbreak reports |  | 46 (100 %) | 70 (100 %) | 60.3 |  |
| Publication (year) |  | 2002 (1998 – 2007) | 2000 (1993 – 2006) |  | 0.075 |
| Start of nosocomial outbreak (year) |  | 1998 (1994 – 2006) | 1996 (1989 – 2001) |  | 0.012 |
| Duration of nosocomial outbreak (weeks) |  | 11 (5 – 35) | 18 (6 – 52) |  | 0.125 |
| **Patients and units involved in nosocomial outbreaks** | | | | | |
| Patient’s age groups | n. s. | 7 (15.2 %) | 14 (20.0 %) | 66.7 | 0.001 |
|  | Adults | 12 (26.1 %) | 38 (54.3 %) | 76.0 |  |
|  | Children | 20 (43.5 %) | 9 (12.9 %) | 31.0 |  |
|  | Adults and Children | 7 (15.2 %) | 9 (12.9 %) | 56.3 |  |
| No. of patients |  | 8 (6 – 14) | 12 (7 – 24) |  | 0.113 |
| No. of patients | >= 10 patients | 16 (34.8 %) | 39 (55.7 %) | 70.9 | 0.027 |
| No. of hematology-oncology patients |  | 8 (4 – 13) | 11 (6 – 20) |  | 0.023 |
| No. of hematology-oncology patients | >= 10 patients | 14 (30.4 %) | 36 (51.4 %) | 72.0 | 0.026 |
| Patient’s age (median) |  | 23 (6 – 55) | 47.75 (34 – 56) |  | 0.063 |
| No. of infected patients |  | 6 (4 – 9) | 8 (5 – 13) |  | 0.052 |
| No. of patients who survived |  | 8 (4 – 13) | 6 (3 – 14) |  | 0.562 |
| No. of patients who died |  | 0 (0 – 0) | 3 (2 – 5) |  | <0.001 |
| No. of units / department |  | 1 (1 – 2) | 1 (1 – 2) |  | 0.307 |
| No. of units / department | n. s. | 9 (19.6 %) | 26 (37.1 %) | 74.3 | 0.077 |
|  | 1 unit / department | 23 (50.0 %) | 32 (45.7 %) | 58.2 |  |
|  | > 1 unit / department | 14 (30.4 %) | 12 (17.1 %) | 46.2 |  |
| No. of oncological units / department |  | 1 (1 – 1) | 1 (1 – 1) |  | 0.746 |
| No. of oncological units / department | n. s. | 9 (19.6 %) | 25 (35.7 %) | 73.5 | 0.158 |
|  | 1 unit / department | 29 (63.0 %) | 37 (52.9 %) | 56.1 |  |
|  | > 1 unit / department | 8 (17.4 %) | 8 (11.4 %) | 50.0 |  |
| **Nosocomial outbreaks in which patients with comorbidities and treatments were involved** | | | | | |
| Solid tumor |  | 25 (54.3 %) | 13 (18.6 %) | 34.2 | <0.001 |
| Hematological-oncological |  | 27 (58.7 %) | 52 (74.3 %) | 65.8 | 0.078 |
| Hematological and not oncological |  | 6 (13.0 %) | 14 (20.0 %) | 70.0 | 0.332 |
| Bone marrow transplantation |  | 10 (21.7 %) | 30 (42.9 %) | 75.0 | 0.019 |
| Antibiotic therapy |  | 9 (19.5 %) | 9 (12.9 %) | 50.0 | 0.329 |
| Chemotherapy |  | 14 (30.4 %) | 17 (24.3 %) | 54.8 | 0.464 |
| Invasive devices |  | 25 (54.3 %) | 6 (8.6 %) | 19.4 | <0.001 |
| **Groups of causative agents in nosocomial outbreaks** | | | | | |
| Bacteria |  | 28 (60.9 %) | 34 (48.6 %) | 54.8 | 0.194 |
| Fungi |  | 8 (17.4 %) | 18 (25.7 %) | 69.2 | 0.293 |
| Viruses |  | 10 (21.7 %) | 17 (24.3 %) | 63.0 | 0.751 |
| **Types of causative agents in nosocomial outbreaks** | | | | | |
| Vancomycin-resistant enterococci |  | 5 (10.9 %) | 13 (18.6 %) | 72.2 | 0.262 |
| Other Gram-positive cocci |  | 0 (0.0 %) | 4 (5.7 %) | 100.0 | 0.099 |
| Other non-fermenting bacteria |  | 11 (23.9 %) | 7 (10.0 %) | 38.9 | 0.043 |
| *Pseudomonas sp.* |  | 8 (17.4 %) | 7 (10.0 %) | 46.7 | 0.246 |
| *Enterobacterales* |  | 5 (10.9 %) | 2 (2.9 %) | 28.6 | 0.076 |
| *Legionella sp.* |  | 0 (0.0 %) | 4 (5.7 %) | 100.0 | 0.099 |
| Spore-forming bacteria |  | 0 (0.0 %) | 2 (2.9 %) | 100.0 | 0.248 |
| *Aspergillus sp.* / *Mucor sp.* |  | 1 (2.2 %) | 11 (15.7 %) | 91.7 | 0.019 |
| Other fungi |  | 4 (8.7 %) | 5 (7.1 %) | 55.6 | 0.760 |
| Respiratory viruses |  | 4 (8.7 %) | 11 (15.7 %) | 73.3 | 0.270 |
| Hepatitis viruses (A/B/C) |  | 4 (8.7 %) | 5 (7.1 %) | 55.6 | 0.760 |
| **Routes of transmission in nosocomial outbreaks** | | | | | |
| Direct contact |  | 5 (10.9 %) | 14 (20.0 %) | 73.7 | 0.194 |
| Indirect contact |  | 33 (71.7 %) | 41 (58.6 %) | 55.4 | 0.149 |
| **Types of infections in nosocomial outbreaks** | | | | | |
| Infected patients only  (no patients with colonizations) |  | 36 (83.7 %) | 51 (76.1 %) | 58.6 | 0.339 |
| Bloodstream infection |  | 28 (60.9 %) | 30 (42.9 %) | 51.7 | 0.058 |
| Urinary tract infection |  | 3 (6.5 %) | 5 (7.1 %) | 62.5 | 0.897 |
| Wound infection |  | 2 (4.3 %) | 4 (5.7 %) | 66.7 | 0.745 |
| Lower respiratory tract infection |  | 4 (8.7 %) | 27 (38.6 %) | 87.1 | <0.001 |
| Vascular catheter infection |  | 10 (21.7 %) | 2 (2.9 %) | 16.7 | 0.001 |
| Gastrointestinal tract infection |  | 3 (6.5 %) | 3 (4.3 %) | 50.0 | 0.595 |
| **Multi-drug resistant pathogens involved in nosocomial outbreaks** | | | | | |
| Multi-drug resistant bacteria |  | 9 (19.6 %) | 20 (28.6 %) | 69.0 | 0.273 |
| Multi-drug resistant Gram-negative rods |  | 4 (8.7 %) | 6 (8.6 %) | 60.0 | 0.981 |
| Vancomycin-resistant enterococci |  | 5 (10.9 %) | 13 (18.6 %) | 72.2 | 0.262 |
| Methicillin-resistant *Staphylococcus aureus* |  | 0 (0.0 %) | 1 (1.4 %) | 100.0 | 0.416 |
| **Sources of nosocomial outbreaks** | | | | | |
| Environment |  | 8 (17.4 %) | 15 (21.4 %) | 65.2 | 0.594 |
| Index person (patient or staff) |  | 7 (15.2 %) | 22 (31.4 %) | 75.9 | 0.049 |
| Drugs i.v. |  | 11 (23.9 %) | 6 (8.6 %) | 35.3 | 0.022 |
| Medical devices |  | 1 (2.2 %) | 6 (8.6 %) | 85.7 | 0.157 |
| Unknown or not reported |  | 19 (41.3 %) | 23 (32.9 %) | 54.8 | 0.354 |
| **Causes of nosocomial outbreaks** | | | | | |
| Human error |  | 7 (15.2 %) | 10 (14.3 %) | 58.8 | 0.890 |
| Technical problem |  | 6 (13.0 %) | 8 (11.4 %) | 57.1 | 0.794 |
| **Infection control measures in nosocomial outbreaks** | | | | | |
| Screening of staff |  | 9 (19.6 %) | 10 (14.3 %) | 52.6 | 0.452 |
| Closure of the ward or department |  | 3 (6.5 %) | 12 (17.1 %) | 80.0 | 0.095 |
| Improvement of staff-to-patient ratio or other changes in staffing |  | 2 (4.3 %) | 6 (8.6 %) | 75.0 | 0.380 |
| Any environmental screening |  | 23 (50.0 %) | 37 (52.9 %) | 61.7 | 0.763 |
| Surfaces |  | 21 (45.7 %) | 30 (42.9 %) | 58.8 | 0.767 |
| Air |  | 2 (4.3 %) | 14 (20.0 %) | 87.5 | 0.017 |
| Water |  | 9 (19.6 %) | 11 (15.7 %) | 55.0 | 0.591 |
| Changes in disinfection and/or sterilization procedures |  | 9 (19.6 %) | 25 (35.7 %) | 73.5 | 0.062 |
| Changes in handling of technical equipment |  | 11 (23.9 %) | 22 (31.4 %) | 66.7 | 0.380 |
| Screening of patients |  | 14 (30.4 %) | 28 (40.0 %) | 66.7 | 0.294 |
| Isolation  (single room or cohort) |  | 10 (21.7%) | 19 (27.1 %) | 65.5 | 0.511 |
| Change of antibiotics or vaccinations |  | 5 (10.9 %) | 18 (25.7 %) | 78.3 | 0.050 |
| Change of antibiotic therapy |  | 2 (4.3 %) | 9 (12.9 %) | 81.8 | 0.126 |
| Change of prophylaxis |  | 2 (4.3 %) | 8 (11.4 %) | 80.0 | 0.184 |
| Vaccination |  | 1 (2.2 %) | 2 (2.9 %) | 66.7 | 0.821 |
| Improvement of hand hygiene |  | 13 (28.3 %) | 16 (22.9 %) | 55.2 | 0.511 |
| Reinforcement of protective clothing |  | 6 (13.0 %) | 12 (17.1 %) | 66.7 | 0.551 |
| Training and education of staff |  | 14 (30.4 %) | 13 (18.6 %) | 48.1 | 0.139 |
| **Weighted cumulative hygiene score (WCHS)** | | | | | |
| WCHS (points) |  | 4 (1 – 7) | 6 (2 – 7) |  | 0.242 |
| WCHS | = 0 points | 9 (19.6 %) | 11 (15.7 %) | 55.0 | 0.591 |
| WCHS | > 4 points | 21 (45.7 %) | 36 (51.4 %) | 63.2 | 0.543 |
| WCHS | > 7 points | 9 (19.6 %) | 17 (24.3 %) | 65.4 | 0.551 |

No., number; Med, Median; IQR, interquartile range; n. s., not specified

**Supplemental File D**

1. Abdallah A, Rowland KE, Schepetiuk SK, To LB, Bardy P (2003) An outbreak of respiratory syncytial virus infection in a bone marrow transplant unit: effect on engraftment and outcome of pneumonia without specific antiviral treatment. Bone Marrow Transplant 32:195-203. doi: 10.1038/sj.bmt.1704116
2. Acosta F, Fernández-Cruz A, Maus SR, Sola-Campoy PJ, Marín M, Cercenado E, Sierra O, Muñoz P, García de Viedma D, Pérez-Lago L. In-depth study of a nosocomial outbreak caused by extensively drug-resistant Pseudomonas aeruginosa using whole genome sequencing coupled with a polymerase chain reaction targeting strain-specific single nucleotide polymorphisms (2020). Am J Epidemiol 189:841-849. doi: 10.1093/aje/kwaa025
3. al-Barrak A, Embil J, Dyck B, Olekson K, Nicoll D, Alfa M, Kabani A. An outbreak of toxin A negative, toxin B positive Clostridium difficile-associated diarrhea in a Canadian tertiary-care hospital (1999. Can Commun Dis Rep 25:65-69
4. Allander T, Gruber A, Naghavi M, Beyene A, Söderström T, Björkholm M, Grillner L, Persson MA. Frequent patient-to-patient transmission of hepatitis C virus in a haematology ward (1995) Lancet 345:603-607. doi: 10.1016/s0140-6736(95)90518-9
5. Anderson K, Morris G, Kennedy H, Croall J, Michie J, Richardson MD, Gibson B. Aspergillosis in immunocompromised paediatric patients: associations with building hygiene, design, and indoor air (1996) Thorax 51:256-261. doi: 10.1136/thx.51.3.256
6. Arnow PM, Chou T, Weil D, Shapiro EN, Kretzschmar C. Nosocomial Legionnaires' disease caused by aerosolized tap water from respiratory devices (1982) J Infect Dis 146:460-467. doi: 10.1093/infdis/146.4.460
7. Aumeran C, Paillard C, Robin F, Kanold J, Baud O, Bonnet R, Souweine B, Traore O. Pseudomonas aeruginosa and Pseudomonas putida outbreak associated with contaminated water outlets in an oncohaematology paediatric unit (2007) J Hosp Infect 65:47-53. doi: 10.1016/j.jhin.2006.08.009
8. Barchiesi F, Caggiano G, Falconi Di Francesco L, Montagna MT, Barbuti S, Scalise G. Outbreak of fungemia due to Candida parapsilosis in a pediatric oncology unit (2004) Diagn Microbiol Infect Dis 49:269-271. doi: 10.1016/j.diagmicrobio.2004.03.011
9. Berk SL, Gage KA, Holtsclaw-Berk SA, Smith JK. Type 8 pneumococcal pneumonia: an outbreak on an oncology ward (1985. South Med J 78:159-161. doi: 10.1097/00007611-198502000-00010
10. Berrouane YF, McNutt LA, Buschelman BJ, Rhomberg PR, Sanford MD, Hollis RJ, Pfaller MA, Herwaldt LA. Outbreak of severe Pseudomonas aeruginosa infections caused by a contaminated drain in a whirlpool bathtub (2000) Clin Infect Dis 31:1331-1337. doi: 10.1086/317501
11. Blot E, Escande MC, Besson D, Barbut F, Granpeix C, Asselain B, Falcou MC, Pouillart P. Outbreak of Clostridium difficile-related diarrhoea in an adult oncology unit: risk factors and microbiological characteristics (2003) J Hosp Infect 53:187-192. doi: 10.1053/jhin.2002.1356
12. Böröcz K, Szilágyi E, Kurcz A, Libisch B, Glatz K, Gacs M. First vancomycin-resistant Enterococcus faecium outbreak reported in Hungary (2005) Euro Surveill 10:E050127.1. doi: 10.2807/esw.10.04.02625-en
13. Boukadida J, de Montalembert M, Gaillard JL, Gobin J, Grimont F, Girault D, Véron M, Berche P. Outbreak of gut colonization by Pseudomonas aeruginosa in immunocompromised children undergoing total digestive decontamination: analysis by pulsed-field electrophoresis (1991) J Clin Microbiol 29:2068-2071. doi: 10.1128/jcm.29.9.2068-2071.1991
14. Boyer PH, Deboscker S, Hernandez C, Ramsheyi M, Schneider P, Foeglé J, Lavigne T. An undiagnosed index case leading to a nosocomial scabies outbreak: how mass single-dose ivermectin treatment can help control a nosocomial epidemic (2018. Infect Control Hosp Epidemiol 39:631-632. doi: 10.1017/ice.2018.31
15. Brazinsky JH, Phillips JE. Pneumocystis pneumonia transmission between patients with lymphoma (1969) JAMA 209:1527. doi: 10.1001/jama.209.10.1527
16. Bressler AM, Kaye KS, LiPuma JJ, Alexander BD, Moore CM, Reller LB, Woods CW. Risk factors for Burkholderia cepacia complex bacteremia among intensive care unit patients without cystic fibrosis: a case-control study (2007) Infect Control Hosp Epidemiol 28:951-958. doi: 10.1086/519177
17. Brillowska-Dabrowska A, Schön T, Pannanusorn S, Lönnbro N, Bernhoff L, Bonnedal J, Haggstrom J, Wistedt A, Fernandez V, Arendrup MC. A nosocomial outbreak of Candida parapsilosis in southern Sweden verified by genotyping (2009) Scand J Infect Dis 41:135-142. doi: 10.1080/00365540802585301
18. Buffington J, Reporter R, Lasker BA, McNeil MM, Lanson JM, Ross LA, Mascola L, Jarvis WR. Investigation of an epidemic of invasive aspergillosis: utility of molecular typing with the use of random amplified polymorphic DNA probes (1994) Pediatr Infect Dis J 13:386-393
19. Burnie J, Carter T, Rigg G, Hodgetts S, Donohoe M, Matthews R. Identification of ABC transporters in vancomycin-resistant Enterococcus faecium as potential targets for antibody therapy (2002) FEMS Immunol Med Microbiol 33: 179-189. doi: 10.1111/j.1574-695X.2002.tb00589.x. PMID: 12110480
20. Burwen DR, Lasker BA, Rao N, Durry E, Padhye AA, Jarvis WR. Invasive aspergillosis outbreak on a hematology-oncology ward (2001) Infect Control Hosp Epidemiol 22:45-48. doi: 10.1086/501826. PMID: 11198023
21. Buttery JP, Alabaster SJ, Heine RG, Scott SM, Crutchfield RA, Bigham A, Tabrizi SN, Garland SM. Multiresistant Pseudomonas aeruginosa outbreak in a pediatric oncology ward related to bath toys (1998) Pediatr Infect Dis J 17:509-513. doi: 10.1097/00006454-199806000-00015
22. Casanova C, Lo Priore E, Egli A, Seth-Smith HMB, Räber L, Ott D, Pflüger V, Droz S, Marschall J, Sommerstein R. Agrobacterium spp. nosocomial outbreak assessment using rapid MALDI-TOF MS based typing, confirmed by whole genome sequencing (2019) Antimicrob Resist Infect Control 8:171. doi: 10.1186/s13756-019-0619-y
23. Centers for Disease Control and Prevention (CDC). Pseudomonas bloodstream infections associated with a heparin/saline flush--Missouri, New York, Texas, and Michigan, 2004-2005 (2005) MMWR Morb Mortal Wkly Rep 54:269-272
24. Chadwick PR, Oppenheim BA, Fox A, Woodford N, Morgenstern GR, Scarffe JH. Epidemiology of an outbreak due to glycopeptide-resistant Enterococcus faecium on a leukaemia unit (1996) J Hosp Infect 34:171-182. doi: 10.1016/s0195-6701(96)90063-8
25. Chang CC, Cheng AC, Devitt B, Hughes AJ, Campbell P, Styles K, Low J, Athan E. Successful control of an outbreak of invasive aspergillosis in a regional haematology unit during hospital construction works (2008) J Hosp Infect 69:33-38. doi: 10.1016/j.jhin.2008.02.010
26. Chapel HM, Christie JM, Peach V, Chapman RW. Five-year follow-up of patients with primary antibody deficiencies following an outbreak of acute hepatitis C (2001) Clin Immunol 99:320-324. doi: 10.1006/clim.2001.5036
27. Chetoui H, Melin P, Struelens MJ, Delhalle E, Nigo MM, De Ryck R, De Mol P. Comparison of biotyping, ribotyping, and pulsed-field gel electrophoresis for investigation of a common-source outbreak of Burkholderia pickettii bacteremia (1997) J Clin Microbiol 35:1398-1403. doi: 10.1128/jcm.35.6.1398-1403.1997
28. Chudy M, Budek I, Keller-Stanislawski B, McCaustland KA, Neidhold S, Robertson BH, Nübling CM, Seitz R, Löwer J. A new cluster of hepatitis A infection in hemophiliacs traced to a contaminated plasma pool (1999) J Med Virol 57:91-99. doi: 10.1002/(sici)1096-9071(199902)57:2<91::aid-jmv1>3.0.co;2-l
29. Chlebicki MP, Ling ML, Koh TH, Hsu LY, Tan BH, How KB, Sng LH, Wang GC, Kurup A, Kang ML, Low JG. First outbreak of colonization and infection with vancomycin-resistant Enterococcus faecium in a tertiary care hospital in Singapore (2006) Infect Control Hosp Epidemiol 27:991-993. doi: 10.1086/507289
30. Cooksey RC, Jhung MA, Yakrus MA, Butler WR, Adékambi T, Morlock GP, Williams M, Shams AM, Jensen BJ, Morey RE, Charles N, Toney SR, Jost KC Jr, Dunbar DF, Bennett V, Kuan M, Srinivasan A. Multiphasic approach reveals genetic diversity of environmental and patient isolates of Mycobacterium mucogenicum and Mycobacterium phocaicum associated with an outbreak of bacteremias at a Texas hospital (2008) Appl Environ Microbiol 74:2480-2487. doi: 10.1128/AEM.02476-07
31. Cornaglia G, Mazzariol A, Lauretti L, Rossolini GM, Fontana R. Hospital outbreak of carbapenem-resistant Pseudomonas aeruginosa producing VIM-1, a novel transferable metallo-beta-lactamase (2000) Clin Infect Dis 31:1119-1125. doi: 10.1086/317448
32. Corvec S, Poirel L, Espaze E, Giraudeau C, Drugeon H, Nordmann P. Long-term evolution of a nosocomial outbreak of Pseudomonas aeruginosa producing VIM-2 metallo-enzyme (2008) J Hosp Infect 68:73-82. doi: 10.1016/j.jhin.2007.10.016
33. del Mar Tomas M, Cartelle M, Pertega S, Beceiro A, Llinares P, Canle D, Molina F, Villanueva R, Cisneros JM, Bou G. Hospital outbreak caused by a carbapenem-resistant strain of Acinetobacter baumannii: patient prognosis and risk-factors for colonisation and infection (2005) Clin Microbiol Infect 11:540-546. doi: 10.1111/j.1469-0691.2005.01184.x
34. Dennis JE, Rhodes KH, Cooney DR, Roberts GD. Nosocomical Rhizopus infection (zygomycosis) in children (1980) J Pediatr 96:824-828. doi: 10.1016/s0022-3476(80)80550-6
35. Denton M, Hawkey PM, Hoy CM, Porter C. Co-existent cross-infection with Streptococcus pneumoniae and group B streptococci on an adult oncology unit (1993) J Hosp Infect 23:271-278. doi: 10.1016/0195-6701(93)90144-o
36. Deplano A, Denis O, Nonhoff C, Rost F, Byl B, Jacobs F, Vankerckhoven V, Goossens H, Struelens MJ. Outbreak of hospital-adapted clonal complex-17 vancomycin-resistant Enterococcus faecium strain in a haematology unit: role of rapid typing for early control (2007) J Antimicrob Chemother 60:849-854. doi: 10.1093/jac/dkm270
37. Dumpis U, Kovalova Z, Jansons J, Cupane L, Sominskaya I, Michailova M, Karayiannis P, Gardovska D, Viazov S, Ross S, Roggendorf M, Pumpens P. An outbreak of HBV and HCV infection in a paediatric oncology ward: epidemiological investigations and prevention of further spread (2003) J Med Virol 69:331-338. doi: 10.1002/jmv.10293
38. Edmond MB, Ober JF, Weinbaum DL, Pfaller MA, Hwang T, Sanford MD, Wenzel RP. Vancomycin-resistant Enterococcus faecium bacteremia: risk factors for infection (1995) Clin Infect Dis 20:1126-1133. doi: 10.1093/clinids/20.5.1126
39. Egan SA, Corcoran S, McDermott H, Fitzpatrick M, Hoyne A, McCormack O, Cullen A, Brennan GI, O'Connell B, Coleman DC. Hospital outbreak of linezolid-resistant and vancomycin-resistant ST80 Enterococcus faecium harbouring an optrA-encoding conjugative plasmid investigated by whole-genome sequencing (2020) J Hosp Infect 105:726-735. doi: 10.1016/j.jhin.2020.05.013
40. El Saleeby CM, Howard SC, Hayden RT, McCullers JA. Association between tea ingestion and invasive Bacillus cereus infection among children with cancer (2004) Clin Infect Dis 39:1536-1539. doi: 10.1086/425358
41. Engelhart S, Krizek L, Glasmacher A, Fischnaller E, Marklein G, Exner M. Pseudomonas aeruginosa outbreak in a haematology-oncology unit associated with contaminated surface cleaning equipment (2002) J Hosp Infect 52:93-98. doi: 10.1053/jhin.2002.1279
42. Erbay A, Kanyilmaz D, Us E, Mumcuoglu I, Karahan ZC, Gozel G, Balaban N, Bodur H. A cluster of Klebsiella pneumoniae bacteremia in a radiation oncology ward (2008) Am J Infect Control 36:678-680. doi: 10.1016/j.ajic.2007.11.004
43. Fisker N, Carlsen NL, Kolmos HJ, Tønning-Sørensen L, Høst A, Christensen PB. Identifying a hepatitis B outbreak by molecular surveillance: a case study (2006) BMJ 332:343-345. doi: 10.1136/bmj.332.7537.343
44. Flynn PM, Williams BG, Hetherington SV, Williams BF, Giannini MA, Pearson TA. Aspergillus terreus during hospital renovation (1993) Infect Control Hosp Epidemiol 14:363-365. doi: 10.1086/646761
45. Fontana C, Favaro M, Pistoia ES, Minelli S, Bossa MC, Altieri A, Testore GP, Leonardis F, Natoli S, Favalli C. The combined use of VIGI@ct (bioMérieux) and fluorescent amplified length fragment polymorphisms in the investigation of potential outbreaks (2007) J Hosp Infect 66:262-268. doi: 10.1016/j.jhin.2007.04.004
46. Garvey MI, Bradley CW, Holden E. Waterborne Pseudomonas aeruginosa transmission in a hematology unit? (2018) Am J Infect Control 46: 383-386. doi: 10.1016/j.ajic.2017.10.013
47. Gerritzen A, Schneweis KE, Brackmann HH, Oldenburg J, Hanfland P, Gerlich WH, Caspari G. Acute hepatitis A in haemophiliacs (1992) Lancet 340:1231-1232. doi: 10.1016/0140-6736(92)92937-b
48. Ghanem G, Hachem RY, Chemaly RF, Dvorak T, Hulten K, Graviss L, Raad II. The role of molecular methods in the prevention of nosocomial methicillin-resistant Staphylococcus aureus clusters in cancer patients (2008) Am J Infect Control 36:656-660. doi: 10.1016/j.ajic.2008.03.007
49. Gillespie TA, Johnson PR, Notman AW, Coia JE, Hanson MF. Eradication of a resistant Pseudomonas aeruginosa strain after a cluster of infections in a hematology/oncology unit (2000) Clin Microbiol Infect 6:125-130. doi: 10.1046/j.1469-0691.2000.00051.x
50. Gooskens J, Jonges M, Claas EC, Meijer A, van den Broek PJ, Kroes AM. Morbidity and mortality associated with nosocomial transmission of oseltamivir-resistant influenza A(H1N1) virus (2009) JAMA 11;301:1042-6. doi: 10.1001/jama.2009.297
51. Granlund M, Carlsson C, Edebro H, Emanuelsson K, Lundholm R. Nosocomial outbreak of vanB2 vancomycin-resistant Enterococcus faecium in Sweden (2006) J Hosp Infect 62:254-256. doi: 10.1016/j.jhin.2005.06.031
52. Gransden WR, Eykyn SJ. Seven cases of bacteremia due to Ochrobactrum anthropic (1992) Clin Infect Dis 15:1068-1069. doi: 10.1093/clind/15.6.1068
53. Gröbner S, Heeg P, Autenrieth IB, Schulte B. Monoclonal outbreak of catheter-related bacteraemia by Ralstonia mannitolilytica on two haemato-oncology wards (2007) J Infect 55:539-544. doi: 10.1016/j.jinf.2007.07.021
54. Gudiol C, Verdaguer R, Angeles Domínguez M, Fernández-Sevilla A, Carratalà J. Outbreak of Legionnaires' disease in immunosuppressed patients at a cancer centre: usefulness of universal urine antigen testing and early levofloxacin therapy (2007) Clin Microbiol Infect 13:1125-1128. doi: 10.1111/j.1469-0691.2007.01805.x
55. Guiguet M, Rekacewicz C, Leclercq B, Brun Y, Escudier B, Andremont A. Effectiveness of simple measures to control an outbreak of nosocomial methicillin-resistant Staphylococcus aureus infections in an intensive care unit (1990) Infect Control Hosp Epidemiol 11:23-26. doi: 10.1086/646074
56. Hahn T, Cummings KM, Michalek AM, Lipman BJ, Segal BH, McCarthy PL Jr. Efficacy of high-efficiency particulate air filtration in preventing aspergillosis in immunocompromised patients with hematologic malignancies (2002) Infect Control Hosp Epidemiol 23:525-531. doi: 10.1086/502101
57. Hanna H, Umphrey J, Tarrand J, Mendoza M, Raad I. Management of an outbreak of vancomycin-resistant enterococci in the medical intensive care unit of a cancer center (2001) Infect Control Hosp Epidemiol 22:217-219. doi: 10.1086/501892
58. Hardie DR, Williamson C. Analysis of the preS1 gene of hepatitis B virus (HBV) to define epidemiologically linked and un-linked infections in South Africa (1997) Arch Virol 142:1829-1841. doi: 10.1007/s007050050200
59. Harvala H, Gaunt E, McIntyre C, Roddie H, Labonte S, Curran E, Othieno R, Simmonds P, Bremner J. Epidemiology and clinical characteristics of parainfluenza virus 3 outbreak in a Haemato-oncology unit (2012) J Infect 65:246-254. doi: 10.1016/j.jinf.2012.04.011
60. Hautala T, Ikäheimo I, Husu H, Säily M, Siitonen T, Koistinen P, Vuopio-Varkila J, Koskela M, Kujala P. A cluster of Candida krusei infections in a haematological unit (2007) BMC Infect Dis 22;7:97. doi: 10.1186/1471-2334-7-97
61. Healey CJ, Sabharwal NK, Daub J, Davidson F, Yap PL, Fleming KA, Chapman RW, Simmonds P, Chapel H. Outbreak of acute hepatitis C following the use of anti-hepatitis C virus--screened intravenous immunoglobulin therapy (1996) Gastroenterology 110:1120-1126. doi: 10.1053/gast.1996.v110.pm8613001
62. Heo ST, Kim SJ, Jeong YG, Bae IG, Jin JS, Lee JC. Hospital outbreak of Burkholderia stabilis bacteraemia related to contaminated chlorhexidine in haematological malignancy patients with indwelling catheters (2008) J Hosp Infect 70:241-245. doi: 10.1016/j.jhin.2008.07.019
63. Herruzo R, Ruiz G, Perez-Blanco V, Gallego S, Mora E, Vizcaino MJ, Omeñaca F. Bla-OXA48 gene microorganisms outbreak, in a tertiary Children's Hospital, Over 3 years (2012-2014): Case Report (2017) Medicine (Baltimore) 96:e7665. doi: 10.1097/MD.0000000000007665
64. Hsueh PR, Teng LJ, Pan HJ, Chen YC, Sun CC, Ho SW, Luh KT. Outbreak of Pseudomonas fluorescens bacteremia among oncology patients (1998) J Clin Microbiol 36:2914-2917. doi: 10.1128/JCM.36.10.2914-2917.1998
65. Hwang YS, Brinton BG, Leonard RB, Blue SR, Woods ML, Carroll KC. Investigation of an outbreak of vancomycin-resistant Enterococcus faecium in a low prevalence university hospital (1998) J Investig Med 46:435-443
66. Imataki O, Makimoto A, Kato S, Bannai T, Numa N, Nukui Y, Morisawa Y, Ishida T, Kami M, Fukuda T, Mori S, Tanosaki R, Takaue Y. Coincidental outbreak of methicillin-resistant Staphylococcus aureus in a hematopoietic stem cell transplantation unit (2006) Am J Hematol 81:664-669. doi: 10.1002/ajh.20668
67. Iwen PC, Davis JC, Reed EC, Winfield BA, Hinrichs SH. Airborne fungal spore monitoring in a protective environment during hospital construction, and correlation with an outbreak of invasive aspergillosis (1994) Infect Control Hosp Epidemiol 15:303-306. doi: 10.1086/646916
68. Jalal H, Bibby DF, Bennett J, Sampson RE, Brink NS, MacKinnon S, Tedder RS, Ward KN. Molecular investigations of an outbreak of parainfluenza virus type 3 and respiratory syncytial virus infections in a hematology unit (2007) J Clin Microbiol 45:1690-1696. doi: 10.1128/JCM.01912-06
69. Jalal H, Bibby DF, Tang JW, Bennett J, Kyriakou C, Peggs K, Cubitt D, Brink NS, Ward KN, Tedder RS. First reported outbreak of diarrhea due to adenovirus infection in a hematology unit for adults (2005) J Clin Microbiol 43:2575-2580. doi: 10.1128/JCM.43.6.2575-2580.2005
70. Januszkiewicz-Lewandowska D, Wysocki J, Rembowska J, Pernak M, Lewandowski K, Nowak T, Nowicka-Kujawska K, Nowak J. Transmission of HCV infection among long-term hospitalized onco-haematological patients (2003) J Hosp Infect 53:120-123. doi: 10.1053/jhin.2002.1301
71. Johnson Z, Thornton L, Tobin A, Lawlor E, Power J, Hillary I, Temperley I. An outbreak of hepatitis A among Irish haemophiliacs (1995) Int J Epidemiol 24:821-828. doi: 10.1093/ije/24.4.821
72. Johnston JM, Latham RH, Meier FA, Green JA, Boshard R, Mooney BR, Edelstein PH. Nosocomial outbreak of Legionnaires' disease: molecular epidemiology and disease control measures (1987) Infect Control 8:53-58. doi: 10.1017/s0195941700067072
73. Jones BL, Clark S, Curran ET, McNamee S, Horne G, Thakker B, Hood J. Control of an outbreak of respiratory syncytial virus infection in immunocompromised adults (2000) J Hosp Infect;44:53-57. doi: 10.1053/jhin.1999.0666
74. Kalai Blagui S, Achour W, Abbassi MS, Bejaoui M, Abdeladhim A, Ben Hassen A. Nosocomial outbreak of OXA-18-producing Pseudomonas aeruginosa in Tunisia (2007) Clin Microbiol Infect 13:794-800. doi: 10.1111/j.1469-0691.2007.01757.x
75. Kampmeier S, Knaack D, Kossow A, Willems S, Schliemann C, Berdel WE, Kipp F, Mellmann A. Weekly screening supports terminating nosocomial transmissions of vancomycin-resistant enterococci on an oncologic ward - a retrospective analysis (2017) Antimicrob Resist Infect Control 16;6:48. doi: 10.1186/s13756-017-0206-z
76. Kapil A, Gulati S, Goel V, Kumar L, Krishnan R, Kochupillai V. Outbreak of nosocomial Acinetobacter baumannii bacteremia in a high risk ward (1998) Med Oncol 15:270-274. doi: 10.1007/BF02787211
77. Kappstein I, Grundmann H, Hauer T, Niemeyer C. Aerators as a reservoir of Acinetobacter junii: an outbreak of bacteraemia in paediatric oncology patients (2000) J Hosp Infect 44:27-30. doi: 10.1053/jhin.1999.0648
78. Kavaliotis J, Loukou I, Trachana M, Gombakis N, Tsagaropoulou-Stigga H, Koliouskas D. Outbreak of varicella in a pediatric oncology unit (1998) Med Pediatr Oncol 31:166-169. doi: 10.1002/(sici)1096-911x(199809)31:3<166::aid-mpo7>3.0.co;2-9
79. Kawalec M, Gniadkowski M, Hryniewicz W. Outbreak of vancomycin-resistant enterococci in a hospital in Gdask, Poland, due to horizontal transfer of different Tn1546-like transposon variants and clonal spread of several strains (2000) J Clin Microbiol 38:3317-3322. doi: 10.1128/JCM.38.9.3317-3322.2000
80. Kawalec M, Gniadkowski M, Zaleska M, Ozorowski T, Konopka L, Hryniewicz W. Outbreak of vancomycin-resistant Enterococcus faecium of the phenotype VanB in a hospital in Warsaw, Poland: probable transmission of the resistance determinants into an endemic vancomycin-susceptible strain (2001) J Clin Microbiol 39:1781-1787. doi: 10.1128/JCM.39.5.1781-1787.2001
81. Kawalec M, Kedzierska J, Gajda A, Sadowy E, Wegrzyn J, Naser S, Skotnicki AB, Gniadkowski M, Hryniewicz W. Hospital outbreak of vancomycin-resistant enterococci caused by a single clone of Enterococcus raffinosus and several clones of Enterococcus faecium (2007) Clin Microbiol Infect 13:893-901. doi: 10.1111/j.1469-0691.2007.01774.x
82. Kedda MA, Kew MC, Cohn RJ, Field SP, Schwyzer R, Song E, Fernandes-Costa F. An outbreak of hepatitis A among South African patients with hemophilia: evidence implicating contaminated factor VIII concentrate as the source (1995) Hepatology 22:1363-1367
83. Kelkar R, Gordon SM, Giri N, Rao K, Ramakrishnan G, Saikia T, Nair CN, Kurkure PA, Pai SK, Jarvis WR, et al. Epidemic iatrogenic Acinetobacter spp. meningitis following administration of intrathecal methotrexate (1989) J Hosp Infect 14:233-243. doi: 10.1016/0195-6701(89)90040-6
84. Kiddy K, Josse E, Griffin N. An outbreak of serious Klebsiella infections related to food blenders (1987) J Hosp Infect 9:191-193. doi: 10.1016/0195-6701(87)90059-4
85. Kilic A, Senses Z, Kurekci AE, Aydogan H, Sener K, Kismet E, Basustaoglu AC. Nosocomial outbreak of Sphingomonas paucimobilis bacteremia in a hemato/oncology unit (2007) Jpn J Infect Dis 60:394-396
86. Kim S, Sung H, Im HJ, Hong SJ, Kim MN. Molecular epidemiological investigation of a nosocomial outbreak of human metapneumovirus infection in a pediatric hemato-oncology patient population (2009) J Clin Microbiol 47:1221-12224. doi: 10.1128/JCM.01959-08
87. Kim T, Jin CE, Sung H, Koo B, Park J, Kim SM, Kim JY, Chong YP, Lee SO, Choi SH, Kim YS, Woo JH, Lee JH, Lee JH, Lee KH, Shin Y, Kim SH. Molecular epidemiology and environmental contamination during an outbreak of parainfluenza virus 3 in a haematology ward (2017) J Hosp Infect 97:403-413. doi: 10.1016/j.jhin.2017.09.003
88. Kline S, Cameron S, Streifel A, Yakrus MA, Kairis F, Peacock K, Besser J, Cooksey RC. An outbreak of bacteremias associated with Mycobacterium mucogenicum in a hospital water supply (2004) Infect Control Hosp Epidemiol 25:1042-1049. doi: 10.1086/502341
89. Kluytmans J, van Leeuwen W, Goessens W, Hollis R, Messer S, Herwaldt L, Bruining H, Heck M, Rost J, van Leeuwen N, et al. Food-initiated outbreak of methicillin-resistant Staphylococcus aureus analyzed by pheno- and genotyping (1995) J Clin Microbiol 33:1121-1128. doi: 10.1128/jcm.33.5.1121-1128.1995
90. Knippschild M, Schmid EN, Uppenkamp M, König E, Meusers P, Brittinger G, Höffkes HG. Infection by Alcaligenes xylosoxidans subsp. xylosoxidans in neutropenic patients (1996) Oncology 53:258-262. doi: 10.1159/000227570
91. Knoll M, Daeschlein G, Okpara-Hofmann J, Klare I, Wilhelms D, Wolf HH, Borneff-Lipp M. Outbreak of vancomycin-resistant enterococci (VRE) in a hematological oncology ward and hygienic preventive measures. A long-term study (2005) Onkologie 28:187-192. doi: 10.1159/000084061
92. Knöll A, Helmig M, Peters O, Jilg W. Hepatitis C virus transmission in a pediatric oncology ward: analysis of an outbreak and review of the literature (2001) Lab Invest 81:251-262. doi: 10.1038/labinvest.3780234
93. Knowles S, Herra C, Devitt E, O'Brien A, Mulvihill E, McCann SR, Browne P, Kennedy MJ, Keane CT. An outbreak of multiply resistant Serratia marcescens: the importance of persistent carriage (2000) Bone Marrow Transplant 25:873-877. doi: 10.1038/sj.bmt.1702218
94. Komatsu M, Kato H, Aihara M, Shimakawa K, Iwasaki M, Nagasaka Y, Fukuda S, Matsuo S, Arakawa Y, Watanabe M, Iwatani Y. High frequency of antibiotic-associated diarrhea due to toxin A-negative, toxin B-positive Clostridium difficile in a hospital in Japan and risk factors for infection (2003) Eur J Clin Microbiol Infect Dis 22:525-529. doi: 10.1007/s10096-003-0992-5
95. Kool JL, Fiore AE, Kioski CM, Brown EW, Benson RF, Pruckler JM, Glasby C, Butler JC, Cage GD, Carpenter JC, Mandel RM, England B, Breiman RF. More than 10 years of unrecognized nosocomial transmission of legionnaires' disease among transplant patients (1998) Infect Control Hosp Epidemiol 19:898-904. doi: 10.1086/647760
96. Kotilainen P, Routamaa M, Peltonen R, Oksi J, Rintala E, Meurman O, Lehtonen OP, Eerola E, Salmenlinna S, Vuopio-Varkila J, Rossi T. Elimination of epidemic methicillin-resistant Staphylococcus aureus from a university hospital and district institutions, Finland (2003) Emerg Infect Dis 9:169-175. doi: 10.3201/eid0902.020233
97. Labarca JA, Leber AL, Kern VL, Territo MC, Brankovic LE, Bruckner DA, Pegues DA. Outbreak of Stenotrophomonas maltophilia bacteremia in allogenic bone marrow transplant patients: role of severe neutropenia and mucositis (2000) Clin Infect Dis 30:195-197. doi: 10.1086/313591
98. Lacey S, Want SV. Pseudomonas pickettii infections in a paediatric oncology unit (1991) J Hosp Infect 17:45-51. doi: 10.1016/0195-6701(91)90076-k
99. Lavery A, Rossney AS, Morrison D, Power A, Keane CT. Incidence and detection of multi-drug-resistant enterococci in Dublin hospitals (1997) J Med Microbiol 46:150-156. doi: 10.1099/00222615-46-2-150
100. L'Ecuyer PB, Diego J, Murphy D, Trovillion E, Jones M, Sahm DF, Fraser VJ. Nosocomial outbreak of gastroenteritis due to Salmonella senftenberg (1996) Clin Infect Dis 23:734-742. doi: 10.1093/clinids/23.4.734
101. Lee W, Burnie JP, Matthews RC, Oppenheim BO, Damani NN. Hospital outbreaks with yeasts (1991) J Hosp Infect 18 Suppl A:237-249. doi: 10.1016/0195-6701(91)90029-8
102. Leenders A, van Belkum A, Janssen S, de Marie S, Kluytmans J, Wielenga J, Löwenberg B, Verbrugh H. Molecular epidemiology of apparent outbreak of invasive aspergillosis in a hematology ward (1996) J Clin Microbiol 34:345-351. doi: 10.1128/jcm.34.2.345-351.1996
103. Lehours P, Rogues AM, Occhialini A, Boulestreau H, Gachie JP, Mégraud F. Investigation of an outbreak due to Alcaligenes xylosoxydans subspecies xylosoxydans by random amplified polymorphic DNA analysis (2002) Eur J Clin Microbiol Infect Dis 21:108-113. doi: 10.1007/s10096-001-0669-x
104. Leruez-Ville M, Chardin-Ouachée M, Neven B, Picard C, Le Guinche I, Fischer A, Rouzioux C, Blanche S. Description of an adenovirus A31 outbreak in a paediatric haematology unit (2006) Bone Marrow Transplant 38:23-28. doi: 10.1038/sj.bmt.1705389
105. Lesens O, Mihaila L, Robin F, Baud O, Romaszko JP, Tourniac O, Constantin JM, Souweine B, Bonnet R, Bouvet A, Beytout J, Traore O, Laurichesse H. Outbreak of colonization and infection with vancomycin-resistant Enterococcus faecium in a French university hospital (2006) Infect Control Hosp Epidemiol 27:984-986. doi: 10.1086/504932
106. Levin AS, Costa SF, Mussi NS, Basso M, Sinto SI, Machado C, Geiger DC, Villares MC, Schreiber AZ, Barone AA, Branchini ML. Candida parapsilosis fungemia associated with implantable and semi-implantable central venous catheters and the hands of healthcare workers (1998) Diagn Microbiol Infect Dis 30:243-249. doi: 10.1016/s0732-8893(98)00006-6
107. Liu Y, Davin-Regli A, Bosi C, Charrel RN, Bollet C. Epidemiological investigation of Pseudomonas aeruginosa nosocomial bacteraemia isolates by PCR-based DNA fingerprinting analysis (1996) J Med Microbiol 45:359-365. doi: 10.1099/00222615-45-5-359
108. Livni G, Yaniv I, Samra Z, Kaufman L, Solter E, Ashkenazi S, Levy I. Outbreak of Mycobacterium mucogenicum bacteraemia due to contaminated water supply in a paediatric haematology-oncology department (2008) J Hosp Infect 70:253-258. doi: 10.1016/j.jhin.2008.07.016
109. Loeb M, Salama S, Armstrong-Evans M, Capretta G, Olde J. A case-control study to detect modifiable risk factors for colonization with vancomycin-resistant enterococci (1999) Infect Control Hosp Epidemiol 20:760-763. doi: 10.1086/501580
110. Loo VG, Bertrand C, Dixon C, Vityé D, DeSalis B, McLean AP, Brox A, Robson HG. Control of construction-associated nosocomial aspergillosis in an antiquated hematology unit (1996) Infect Control Hosp Epidemiol 17:360-364. doi: 10.1086/647317
111. Loosveld OJ, Schouten HC, Pasmans HM, van Engelshoven JM, Blijham GH. Invasive Aspergillus infections in patients with a malignancy: description of an outbreak and overview of the literature (1992) Neth J Med 40:62-68
112. Lyytikäinen O, Golovanova V, Kolho E, Ruutu P, Sivonen A, Tiittanen L, Hakanen M, Vuopio-Varkila J. Outbreak caused by tobramycin-resistant Pseudomonas aeruginosa in a bone marrow transplantation unit (2001) Scand J Infect Dis 33:445-449. doi: 10.1080/00365540152029918
113. Macedo de Oliveira A, White KL, Leschinsky DP, Beecham BD, Vogt TM, Moolenaar RL, Perz JF, Safranek TJ. An outbreak of hepatitis C virus infections among outpatients at a hematology/oncology clinic (2005) Ann Intern Med 142:898-902. doi: 10.7326/0003-4819-142-11-200506070-00007
114. Magee JT, Hindmarch JM, Nicol CD. Typing of Streptococcus pyogenes by pyrolysis mass spectrometry (1991) J Med Microbiol 35:304-306. doi: 10.1099/00222615-35-5-304
115. Mahoney DH Jr, Steuber CP, Starling KA, Barrett FF, Goldberg J, Fernbach DJ. An outbreak of aspergillosis in children with acute leukemia (1979) J Pediatr 95:70-72. doi: 10.1016/s0022-3476(79)80086-4
116. Mailliard ME, Capadano ME, Hrnicek MJ, Gilroy RK, Gulizia JM. Outcomes of a patient-to-patient outbreak of genotype 3a hepatitis C (2009) Hepatology 50:361-368. doi: 10.1002/hep.22992
117. Maragakis LL, Chaiwarith R, Srinivasan A, Torriani FJ, Avdic E, Lee A, Ross TR, Carroll KC, Perl TM. Sphingomonas paucimobilis bloodstream infections associated with contaminated intravenous fentanyl (2009) Emerg Infect Dis 15:12-18. doi: 10.3201/eid1501.081054
118. Marroni M, Pasticci MB, Pantosti A, Colozza MA, Stagni G, Tonato M. Outbreak of infusion-related septicemia by Ralstonia pickettii in the Oncology Department (2003) Tumori 89:575-576. doi: 10.1177/030089160308900528
119. Mattner F, Sykora KW, Meissner B, Heim A. An adenovirus type F41 outbreak in a pediatric bone marrow transplant unit: analysis of clinical impact and preventive strategies (2008) Pediatr Infect Dis J 27:419-424. doi: 10.1097/INF.0b013e3181658c46
120. McCarthy KM, Van Nierop W, Duse A, Von Gottberg A, Kassel M, Perovic O, Smego R. Control of an outbreak of vancomycin-resistant Enterococcus faecium in an oncology ward in South Africa: effective use of limited resources (2000) J Hosp Infect 44:294-300. doi: 10.1053/jhin.1999.0696
121. Meyers JD, Huff JC, Holmes KK, Thomas ED, Bryan JA. Parenterally transmitted hepatitis A associated with platelet transfusions. Epidemiologic study of an outbreak in a marrow transplantation center (1974) Ann Intern Med 81:145-151. doi: 10.7326/0003-4819-81-2-145
122. Meyers JD, MacQuarrie MB, Merigan TC, Jennison MH. Nosocomial varicella. Part I: outbreak in oncology patients at a children's hospital (1979) West J Med 130:196-199
123. Micol JB, de Botton S, Guieze R, Coiteux V, Darre S, Dessein R, Leroy O, Yakoub-Agha I, Quesnel B, Bauters F, Beaucaire G, Alfandari S. An 18-case outbreak of drug-resistant Pseudomonas aeruginosa bacteriemia in hematology patients (2006) Haematologica 91:1134-1138
124. Montecalvo MA, Horowitz H, Gedris C, Carbonaro C, Tenover FC, Issah A, Cook P, Wormser GP. Outbreak of vancomycin-, ampicillin-, and aminoglycoside-resistant Enterococcus faecium bacteremia in an adult oncology unit (1994) Antimicrob Agents Chemother 38:1363-1367. doi: 10.1128/AAC.38.6.1363
125. Moriguchi N, Itahashi Y, Tabata N, Yamazumi T, Furuta I, Shibata N, Arakawa Y, Miyata H. Outbreak of CTX-M-3-type extended-spectrum beta-lactamase-producing Enterobacter cloacae in a pediatric ward (2007) J Infect Chemother 13:263-266. doi: 10.1007/s10156-007-0526-7
126. Moschovi MA, Theodoridou M, Papaevangelou V, Youroukos S, Nitsa A, Matsiota P, Tzortzatou-Stathopoulou F. Outbreak of enteroviral infection in a pediatric hematology-oncology unit (2002) Clin Infect Dis 34:1269-1272. doi: 10.1086/339958
127. Muta T, Tsuruta N, Seki Y, Ota R, Suzuki S, Shibata N, Kato K, Eto T, Gondo H, Arakawa Y. A nosocomial outbreak due to novel CTX-M-2-producing strains of Citrobacter koseri in a hematological ward (2006) Jpn J Infect Dis 59:69-71
128. Myoken Y, Sugata T, Fujita Y, Kyo T, Fujihara M, Kohara T, Katsu M, Mikami Y. Molecular epidemiology of invasive stomatitis due to Aspergillus flavus in patients with acute leukemia (2003) J Oral Pathol Med 32:215-218. doi: 10.1034/j.1600-0714.2003.00080.x
129. Nabeya D, Kinjo T, Parrott GL, Uehara A, Motooka D, Nakamura S, Nahar S, Nakachi S, Nakamatsu M, Maeshiro S, Haranaga S, Tateyama M, Tomoyose T, Masuzaki H, Horii T, Fujita J. The clinical and phylogenetic investigation for a nosocomial outbreak of respiratory syncytial virus infection in an adult hemato-oncology unit (2017) J Med Virol 89:1364-1372. doi: 10.1002/jmv.24800.
130. Naumovski L, Quinn JP, Miyashiro D, Patel M, Bush K, Singer SB, Graves D, Palzkill T, Arvin AM. Outbreak of ceftazidime resistance due to a novel extended-spectrum beta-lactamase in isolates from cancer patients (1992) Antimicrob Agents Chemother 36:1991-1996. doi: 10.1128/AAC.36.9.1991.
131. Nolte FS, Conlin CA, Roisin AJ, Redmond SR. Plasmids as epidemiological markers in nosocomial Legionnaires' disease (1984) J Infect Dis 149:251-256. doi: 10.1093/infdis/149.2.251.
132. Nourse C, Murphy H, Byrne C, O'Meara A, Breatnach F, Kaufmann M, Clarke A, Butler K. Control of a nosocomial outbreak of vancomycin resistant Enterococcus faecium in a paediatric oncology unit: risk factors for colonization (1998) Eur J Pediatr 157:20-27. doi: 10.1007/s004310050760.
133. Nucci M, Akiti T, Barreiros G, Silveira F, Revankar SG, Wickes BL, Sutton DA, Patterson TF. Nosocomial outbreak of Exophiala jeanselmei fungemia associated with contamination of hospital water (2002) Clin Infect Dis 34:1475-1480. doi: 10.1086/340344.
134. Oh HS, Kim EC, Oh MD, Choe KW. Outbreak of vancomycin resistant enterococcus in a hematology/oncology unit in a Korean University Hospital, and risk factors related to patients, staff, hospital care and facilities (2004) Scand J Infect Dis 36:790-794. doi: 10.1080/00365540410021117.
135. Olver WJ, James SA, Lennard A, Galloway A, Roberts IN, Boswell TC, Russell NH. Nosocomial transmission of Saccharomyces cerevisiae in bone marrow transplant patients (2002) J Hosp Infect 52:268-272. doi: 10.1053/jhin.2002.1314.
136. Oren I, Haddad N, Finkelstein R, Rowe JM. Invasive pulmonary aspergillosis in neutropenic patients during hospital construction: before and after chemoprophylaxis and institution of HEPA filters (2001) Am J Hematol 66:257-262. doi: 10.1002/ajh.1054.
137. Oren I, Zuckerman T, Avivi I, Finkelstein R, Yigla M, Rowe JM. Nosocomial outbreak of Legionella pneumophila serogroup 3 pneumonia in a new bone marrow transplant unit: evaluation, treatment and control (2002) Bone Marrow Transplant 30:175-179. doi: 10.1038/sj.bmt.1703628.
138. Orth B, Frei R, Itin PH, Rinaldi MG, Speck B, Gratwohl A, Widmer AF. Outbreak of invasive mycoses caused by Paecilomyces lilacinus from a contaminated skin lotion (1996) Ann Intern Med 125:799-806. doi: 10.7326/0003-4819-125-10-199611150-00003.
139. Ozdamar M, Turkoglu S. A nosocomial scabies outbreak originating from immunocompromised transplant patients in Turkey: Upholstery as a possible cause (2020) Transpl Infect Dis 22:e13284. doi: 10.1111/tid.13284.
140. Ozkocaman V, Ozcelik T, Ali R, Ozkalemkas F, Ozkan A, Ozakin C, Akalin H, Ursavas A, Coskun F, Ener B, Tunali A. Bacillus spp. among hospitalized patients with haematological malignancies: clinical features, epidemics and outcomes (2006) J Hosp Infect 64:169-76. doi: 10.1016/j.jhin.2006.05.014.
141. Silva CP, Amarante JM, Lacerda RA, Biancalana ML. Klebsiella pneumoniae outbreak in a cancer unit of a general hospital: predisposing factors and evaluation of the impact of intervention measures (2005) Braz J Infect Dis 9:225-230. doi: 10.1590/s1413-86702005000300005.
142. Pegues DA, Carson LA, Anderson RL, Norgard MJ, Argent TA, Jarvis WR, Woernle CH. Outbreak of Pseudomonas cepacia bacteremia in oncology patients (1993) Clin Infect Dis 16:407-411. doi: 10.1093/clind/16.3.407.
143. Pendle S, Jelfs P, Olma T, Su Y, Gilroy N, Gilbert GL. Difficulties in detection and identification of Enterococcus faecium with low-level inducible resistance to vancomycin, during a hospital outbreak (2008) Clin Microbiol Infect 14:853-857. doi: 10.1111/j.1469-0691.2008.02052.x.
144. Penzak SR, Gubbins PO, Stratton SL, Anaissie EJ. Investigation of an outbreak of gram-negative bacteremia among hematology-oncology outpatients (2000) Infect Control Hosp Epidemiol 21:597-599. doi: 10.1086/501810.
145. Perola O, Nousiainen T, Suomalainen S, Aukee S, Kärkkäinen UM, Kauppinen J, Ojanen T, Katila ML. Recurrent Sphingomonas paucimobilis -bacteraemia associated with a multi-bacterial water-borne epidemic among neutropenic patients (2002) J Hosp Infect 50:196-201. doi: 10.1053/jhin.2001.1163.
146. Petrosillo N, Ippolito G, Solforosi L, Varaldo PE, Clementi M, Manzin A. Molecular epidemiology of an outbreak of fulminant hepatitis B (2000) J Clin Microbiol 38:2975-2981. doi: 10.1128/JCM.38.8.2975-2981.2000.
147. Piralla A, Percivalle E, Di Cesare-Merlone A, Locatelli F, Gerna G. Multicluster nosocomial outbreak of parainfluenza virus type 3 infection in a pediatric oncohematology unit: a phylogenetic study (2009) Haematologica 94:833-839. doi: 10.3324/haematol.2008.003319.
148. Plainvert C, Longo M, Seringe E, Saintpierre B, Sauvage E, Ma L, Beghain J, Dmytruk N, Collobert G, Hernandez E, Manuel C, Astagneau P, Glaser P, Ariey F, Poyart C, Fouet A. A clone of the emergent Streptococcus pyogenes emm89 clade responsible for a large outbreak in a post-surgery oncology unit in France (2018) Med Microbiol Immunol 207:287-296. doi: 10.1007/s00430-018-0546-1.
149. Poirel L, Lebessi E, Castro M, Fèvre C, Foustoukou M, Nordmann P. Nosocomial outbreak of extended-spectrum beta-lactamase SHV-5-producing isolates of Pseudomonas aeruginosa in Athens, Greece (2004) Antimicrob Agents Chemother 48:2277-9. doi: 10.1128/AAC.48.6.2277-2279.2004.
150. Posteraro B, Bruno S, Boccia S, Ruggiero A, Sanguinetti M, Romano Spica V, Ricciardi G, Fadda G. Candida parapsilosis bloodstream infection in pediatric oncology patients: results of an epidemiologic investigation (2004) Infect Control Hosp Epidemiol 25:641-645. doi: 10.1086/502454.
151. Richet H, Escande MC, Marie JP, Zittoun R, Lagrange PH. Epidemic Pseudomonas aeruginosa serotype O16 bacteremia in hematology-oncology patients (1989) J Clin Microbiol 27:1992-1996. doi: 10.1128/jcm.27.9.1992-1996.1989.
152. Roberts LA, Collignon PJ, Cramp VB, Alexander S, McFarlane AE, Graham E, Fuller A, Sinickas V, Hellyar A. An Australia-wide epidemic of Pseudomonas pickettii bacteraemia due to contaminated "sterile" water for injection (1990) Med J Aust 152:652-655. doi: 10.5694/j.1326-5377.1990.tb125422.x.
153. Robin C, Alanio A, Gits-Muselli M, la Martire G, Schlemmer F, Botterel F, Angebault C, Leclerc M, Beckerich F, Redjoul R, Pautas C, Toma A, Maury S, Bretagne S, Cordonnier C. Molecular demonstration of a Pneumocystis outbreak in stem cell transplant patients: evidence for transmission in the daycare center (2017) Front Microbiol 8:700. doi: 10.3389/fmicb.2017.00700.
154. Rogers M, Weinstock DM, Eagan J, Kiehn T, Armstrong D, Sepkowitz KA. Rotavirus outbreak on a pediatric oncology floor: possible association with toys (2000) Am J Infect Control 28:378-380. doi: 10.1067/mic.2000.109908.
155. Rudin JE, Wing EJ. A comparative study of Legionella micdadei and other nosocomial acquired pneumonia (1984) Chest 86:675-680. doi: 10.1378/chest.86.5.675.
156. Ruutu P, Valtonen V, Elonen E, Volin L, Tukiainen P, Ruutu T. Invasive pulmonary aspergillosis: a diagnostic and therapeutic problem. Clinical experience with eight haematologic patients (1987) Scand J Infect Dis 19:569-575. doi: 10.3109/00365548709032424.
157. Salgado CD, Mauldin PD, Fogle PJ, Bosso JA. Analysis of an outbreak of Clostridium difficile infection controlled with enhanced infection control measures (2009) Am J Infect Control 37:458-464. doi: 10.1016/j.ajic.2008.11.010.
158. Sample ML, Gravel D, Oxley C, Toye B, Garber G, Ramotar K. An outbreak of vancomycin-resistant enterococci in a hematology-oncology unit: control by patient cohorting and terminal cleaning of the environment (2002) Infect Control Hosp Epidemiol 23:468-470. doi: 10.1086/502088.
159. Samuel J, Coutinho H, Galloway A, Rennison C, Kaufmann ME, Woodford N. Glycopeptide-resistant Enterococcus raffinosus in a haematology unit: an unusual cause of a nosocomial outbreak (2008) J Hosp Infect 70:294-296. doi: 10.1016/j.jhin.2008.06.029.
160. Schuster F, Graubner UB, Schmid I, Weiss M, Belohradsky BH. Vancomycin-resistant-enterococci--colonization of 24 patients on a pediatric oncology unit (1998) Klin Padiatr 210:261-263. doi: 10.1055/s-2008-1043889.
161. Sebert ME, Manning ML, McGowan KL, Alpern ER, Bell LM. An outbreak of Serratia marcescens bacteremia after general anesthesia (2002) Infect Control Hosp Epidemiol 23:733-739. doi: 10.1086/502003.
162. Sekiguchi J, Teruya K, Horii K, Kuroda E, Konosaki H, Mizuguchi Y, Araake M, Kawana A, Yoshikura H, Kuratsuji T, Miyazaki H, Kirikae T. Molecular epidemiology of outbreaks and containment of drug-resistant Pseudomonas aeruginosa in a Tokyo hospital (2007) J Infect Chemother 13:418-422. doi: 10.1007/s10156-007-0560-5.
163. Shaw PJ. Suspected infection in children with cancer (2002) J Antimicrob Chemother 49 Suppl 1:63-67. doi: 10.1093/jac/49.suppl_1.63.
164. Shetty A, McLauchlin J, Grant K, O'Brien D, Howard T, Davies EM. Outbreak of Listeria monocytogenes in an oncology unit associated with sandwiches consumed in hospital (2009) J Hosp Infect 72:332-336. doi: 10.1016/j.jhin.2009.01.012.
165. Shin JH, Kook H, Shin DH, Hwang TJ, Kim M, Suh SP, Ryang DW. Nosocomial cluster of Candida lipolytica fungemia in pediatric patients (2000) Eur J Clin Microbiol Infect Dis 19:344-349. doi: 10.1007/s100960050491.
166. Silini E, Locasciulli A, Santoleri L, Gargantini L, Pinzello G, Montillo M, Foti L, Lisa A, Orfeo N, Magliano E, Nosari A, Morra E. Hepatitis C virus infection in a hematology ward: evidence for nosocomial transmission and impact on hematologic disease outcome (2002) Haematologica 87:1200-1208.
167. Simon A, Schildgen O, Maria Eis-Hübinger A, Hasan C, Bode U, Buderus S, Engelhart S, Fleischhack G. Norovirus outbreak in a pediatric oncology unit (2006) Scand J Gastroenterol 41:693-699. doi: 10.1080/00365520500421694.
168. Singer C, Armstrong D, Rosen PP, Schottenfeld D. Pneumocystis carinii pneumonia: a cluster of eleven cases (1975) Ann Intern Med 82:772-777. doi: 10.7326/0003-4819-82-6-722.
169. Spada E, Sagliocca L, Sourdis J, Garbuglia AR, Poggi V, De Fusco C, Mele A. Use of the minimum spanning tree model for molecular epidemiological investigation of a nosocomial outbreak of hepatitis C virus infection (2004) J Clin Microbiol 42:4230-4236. doi: 10.1128/JCM.42.9.4230-4236.2004.
170. Stingeni L, Tramontana M, Principato M, Moretta I, Principato S, Bianchi L, Hansel K. Nosocomial outbreak of crusted scabies in immunosuppressed patients caused by Sarcoptes scabiei var. canis (2020) Br J Dermatol 182:498-500. doi: 10.1111/bjd.18539.
171. Stephenson JR, Heard SR, Richards MA, Tabaqchali S. Gastrointestinal colonization and septicaemia with Pseudomonas aeruginosa due to contaminated thymol mouthwash in immunocompromised patients (1985) J Hosp Infect 6:369-378. doi: 10.1016/0195-6701(85)90053-2.
172. Szomor KN, Dencs A, Tóth G, Kovács GM, Saleh Ali Y, Berencsi G, Takács M. Variability of the PreS1/PreS2/S regions of hepatitis B virus in Hungary (2007) Arch Virol 152:697-704. doi: 10.1007/s00705-006-0902-0.
173. Takigawa K, Fujita J, Negayama K, Yamagishi Y, Yamaji Y, Ouchi K, Yamada K, Abe M, Nakazawa T, Kawanishi K, et al. Nosocomial outbreak of Pseudomonas cepacia respiratory infection in immunocompromised patients associated with contaminated nebulizer devices (1993) Kansenshogaku Zasshi 67:1115-1125. doi: 10.11150/kansenshogakuzasshi1970.67.1115.
174. Taneja N, Khurana S, Trehan A, Marwaha RK, Sharma M. An outbreak of hospital acquired diarrhea due to Aeromonas sobria (2004) Indian Pediatr 41:912-916.
175. Thio CL, Smith D, Merz WG, Streifel AJ, Bova G, Gay L, Miller CB, Perl TM. Refinements of environmental assessment during an outbreak investigation of invasive aspergillosis in a leukemia and bone marrow transplant unit (2000) Infect Control Hosp Epidemiol 21:18-23. doi: 10.1086/501691.
176. Tedder RS, Zuckerman MA, Goldstone AH, Hawkins AE, Fielding A, Briggs EM, Irwin D, Blair S, Gorman AM, Patterson KG, et al. Hepatitis B transmission from contaminated cryopreservation tank (1995) Lancet 346:137-40. doi: 10.1016/s0140-6736(95)91207-x.
177. Thuler LC, Faivichenco S, Velasco E, Martins CA, Nascimento CR, Castilho IA. Fungaemia caused by Hansenula anomala--an outbreak in a cancer hospital (1997) Mycoses 40:193-196. doi: 10.1111/j.1439-0507.1997.tb00213.x.
178. Thuler LC, Velasco E, de Souza Martins CA, de Faria LM, da Fonseca NP, Dias LM, Gonçalves VM. An outbreak of Bacillus species in a cancer hospital (1998) Infect Control Hosp Epidemiol 19:856-858. doi: 10.1086/647746.
179. Timmers GJ, van der Zwet WC, Simoons-Smit IM, Savelkoul PH, Meester HH, Vandenbroucke-Grauls CM, Huijgens PC. Outbreak of vancomycin-resistant Enterococcus faecium in a haematology unit: risk factor assessment and successful control of the epidemic (2002) Br J Haematol 116:826-833. doi: 10.1046/j.0007-1048.2002.03339.x.
180. Velasco D, Perez S, Angeles Domínguez M, Villanueva R, Bou G. Description of a nosocomial outbreak of infection caused by a vanA-containing strain of Enterococcus faecalis in La Coruna, Spain (2004) J Antimicrob Chemother 53:892-893. doi: 10.1093/jac/dkh190.
181. Vianelli N, Giannini MB, Quarti C, Bucci Sabattini MA, Fiacchini M, de Vivo A, Graldi P, Galli S, Nanetti A, Baccarani M, Ricci P. Resolution of a Pseudomonas aeruginosa outbreak in a hematology unit with the use of disposable sterile water filters (2006) Haematologica 91:983-985.
182. Watson JT, Jones RC, Siston AM, Fernandez JR, Martin K, Beck E, Sokalski S, Jensen BJ, Arduino MJ, Srinivasan A, Gerber SI. Outbreak of catheter-associated Klebsiella oxytoca and Enterobacter cloacae bloodstream infections in an oncology chemotherapy center (2005) Arch Intern Med 165:2639-2643. doi: 10.1001/archinte.165.22.2639.
183. Weems JJ Jr, Davis BJ, Tablan OC, Kaufman L, Martone WJ. Construction activity: an independent risk factor for invasive aspergillosis and zygomycosis in patients with hematologic malignancy (1987) Infect Control 8:71-75. doi: 10.1017/s0195941700067114.
184. Widell A, Christensson B, Wiebe T, Schalén C, Hansson HB, Allander T, Persson MA. Epidemiologic and molecular investigation of outbreaks of hepatitis C virus infection on a pediatric oncology service (1999) Ann Intern Med 130:130-134. doi: 10.7326/0003-4819-130-2-199901190-00007.
185. Worth LJ, Thursky KA, Seymour JF, Slavin MA. Vancomycin-resistant Enterococcus faecium infection in patients with hematologic malignancy: patients with acute myeloid leukemia are at high-risk (2007) Eur J Haematol 79:226-233. doi: 10.1111/j.1600-0609.2007.00911.x.
186. Yetkin G, Otlu B, Cicek A, Kuzucu C, Durmaz R. Clinical, microbiologic, and epidemiologic characteristics of Pseudomonas aeruginosa infections in a University Hospital, Malatya, Turkey (2006) Am J Infect Control 34:188-192. doi: 10.1016/j.ajic.2005.11.010.
187. Yoo JH, Lee DG, Choi SM, Choi JH, Shin WS, Kim M, Yong D, Lee K, Min WS, Kim CC. Vancomycin-resistant enterococcal bacteremia in a hematology unit: molecular epidemiology and analysis of clinical course (2005) J Korean Med Sci 20:169-176. doi: 10.3346/jkms.2005.20.2.169.
188. Yue MC, Collins JT, Subramoniapillai E, Kennedy GA. Successful use of oseltamivir prophylaxis in managing a nosocomial outbreak of influenza A in a hematology and allogeneic stem cell transplant unit (2017) Asia Pac J Clin Oncol 13:37-43. doi: 10.1111/ajco.12565.
189. Zanella RC, Brandileone MC, Bokermann S, Almeida SC, Valdetaro F, Vitório F, Moreira Mde F, Villins M, Salomão R, Pignatari AC. Phenotypic and genotypic characterization of VanA Enterococcus isolated during the first nosocomial outbreak in Brazil (2003) Microb Drug Resist 9:283-291. doi: 10.1089/107662903322286490.
190. Zambon M, Bull T, Sadler CJ, Goldman JM, Ward KN. Molecular epidemiology of two consecutive outbreaks of parainfluenza 3 in a bone marrow transplant unit (1998) J Clin Microbiol 36:2289-2293. doi: 10.1128/JCM.36.8.2289-2293.1998.
